# Suspension of Amorphous Calcium Phosphate Nanoparticles Impact Commitment of Human Adipose-Derived Stem Cells In Vitro

**DOI:** 10.3390/biology10070675

**Published:** 2021-07-16

**Authors:** Petra Wolint, Lukas Näf, Désirée Schibler, Nora Hild, Wendelin J. Stark, Pietro Giovanoli, Maurizio Calcagni, Johanna Buschmann

**Affiliations:** 1 Division of Plastic and Hand Surgery, University Hospital Zurich, 8091 Zurich, Switzerland; Petra.Wolint@usz.ch (P.W.); luknaef@gmail.com (L.N.); desiree.schibler@uzh.ch (D.S.); Pietro.Giovanoli@usz.ch (P.G.); maurizio.calcagni@usz.ch (M.C.); 2 Institute for Chemical and Bioengineering, Department of Chemistry and Applied Biosciences, ETH Zurich, 8093 Zurich, Switzerland; nora.hild@alumni.ethz.ch (N.H.); wendelin.stark@chem.ethz.ch (W.J.S.)

**Keywords:** amorphous calcium phosphate, nanoparticle, adipose-derived stem cells, osteogenesis, angiogenesis, chondrogenesis, adipogenesis, hydroxyapatite, calcium ion, phosphate ion

## Abstract

**Simple Summary:**

Calcium phosphate is an important component in natural bone. Bone defects caused by trauma or resection of tumors demand bone substitutes to close the defect. One viable option is to use a scaffold material seeded with stem cells. Stem cells can differentiate towards bone cells (osteoblasts), among other cell types. To trigger stem cells towards the osteoblast type of cell, particular supplementation of the culture medium is needed. This study tests whether calcium phosphate nanoparticles can induce a commitment towards the osteoblast type of cell. In addition, we test for other commitments, such as endothelial cell, chondrocyte, or adipocyte commitment. After 1 or 2 weeks, with either 5 or 50 µg/mL nanoparticles in the culture medium, gene expression is analyzed. We find a significant increase of two specific bone marker genes after two weeks in 50 µg/mL nanoparticles compared to 5 µg/mL for two out of three tested human donors of adipose-derived stem cells. Moreover, endothelial cell commitment is also induced. Hence, such nanoparticles have the potential to trigger osteogenic and endothelial cell commitment.

**Abstract:**

Amorphous calcium phosphate (aCaP) nanoparticles may trigger the osteogenic commitment of adipose-derived stem cells (ASCs) in vitro. The ASCs of three human donors are investigated using basal culture medium DMEM to either 5 or 50 µg/mL aCaP nanoparticles suspension (control: no nanoparticles). After 7 or 14 days, stem cell marker genes, as well as endothelial, osteogenic, chondrogenic, and adipogenic genes, are analyzed by qPCR. Free calcium and phosphate ion concentrations are assessed in the cell culture supernatant. After one week and 5 µg/mL aCaP, downregulation of osteogenic markers ALP and Runx2 is found, and averaged across the three donors. Our results show that after two weeks, ALP is further downregulated, but Runx2 is upregulated. Endothelial cell marker genes, such as CD31 and CD34, are upregulated with 50 µg/mL aCaP and a 2-week exposure. Inter-donor variability is high: Two out of three donors show a significant upregulation of ALP and Runx2 at day 14 with 50 µg/mL aCaP compared to 5 µg/mL aCaP. Notably, all changes in stem cell commitment are obtained in the absence of an osteogenic medium. While the chemical composition of the culture medium and the saturation status towards calcium phosphate phases remain approximately the same for all conditions, gene expression of ASCs changes considerably. Hence, aCaP nanoparticles show the potential to trigger osteogenic and endothelial commitment in ASCs.

## 1. Introduction

Calcium phosphate (Ca_3_(PO_4_)_2_) is a biomaterial that is often used in orthopedic surgery, either in the form of cement [1,2], ceramic [3], or coating of titanium implants [4]. Nanoparticles of calcium phosphate may originate from mechanical abrasion of implant materials, as well as through degradation of larger entities. Nanoscopic calcium phosphate has been reported to represent no health risk to the human body because calcium phosphate nanoparticles are easily resorbed and dissolved by macrophages [5].

Calcium phosphate nanoparticles have been chemically produced by wet- and dry-based methods, such as precipitation or flame spray pyrolysis [6]. Furthermore, calcium phosphate phases differ in terms of solubility, where crystalline forms like alpha-tricalcium phosphate (α-TCP) and beta-tricalcium phosphate (β-TCP) are less soluble in aqueous solution than amorphous tricalcium phosphate (aCaP) [7], resulting in higher free calcium (Ca^2+^) and phosphate ion (Pi) concentrations in equilibrium with solid aCaP. Amorphous calcium phosphate has gained a lot of attention, due to its bioactivity; it easily transforms to hydroxyapatite (HAp) [8], the main inorganic component of natural bone [9]. Like this, aCaP is an interesting material for bone tissue engineering purposes, particularly if combined with an organic phase to give nanocomposites, such as PLGA/aCaP [10,11,12].

During the last two decades, adipose tissue-derived mesenchymal stem cells (ASCs) have been proven attractive for cellular therapy and bone tissue engineering purposes, because they are easily harvested and available in quite high amounts compared to other sources [13,14,15,16]. Their tri-lineage differentiation potential makes them sensitive towards corresponding culture medium supplementation, with osteogenesis, chondrogenesis, and adipogenic differentiation belonging to the essential stem cell criteria defined by Dominici et al. [17]. For in vitro osteogenic differentiation, a phosphate source should be supplied to the medium to enable the alkaline phosphatase (ALP) of ASCs to produce Pi ions which take part in mineralization and the formation of HAp. Usually, β-glycerophosphate is used for this purpose; however, critical comments about its high and non-physiological concentration (10 mM), as well as the wide fluctuations of the Pi ions during differentiation experiments, have been raised [18]. Therefore, other phosphate sources have been suggested, such as sodium hydrogen phosphate buffer [18,19] or polyphosphate [20].

As various calcium phosphate-based biomaterials have shown to trigger osteogenesis in ASCs even in the absence of osteogenic culture medium (i.e., without β-glycerophosphate or other further phosphate sources) [21,22], the question arises if aCaP nanoparticles suspended in basal culture medium Dubelcco’s Modified Eagle’s Medium (DMEM) [23] can evoke an osteogenic commitment. The novelty of our approach lies in using *amorphous* calcium phosphate nanoparticles, not crystalline nanoparticles; and in using very low concentrations of suspended particles, such as 5 and 50 μg/mL, respectively. The addition of a small number of aCaP nanoparticles to DMEM with a subsequent ultrasound step to suspend them is easy to perform. The potential impact of such nanoparticles lies in their prospective ability to trigger osteogenic commitment in stem cells, to provide a Pi source, and to transform into HAp. Therefore, we exposed ASCs of three human donors to aCaP nanoparticles in vitro and assessed the gene expression at 1 and 2 weeks for two different concentrations; 5 and 50 μg/mL, respectively, where cell viability was guaranteed in contrast to 500 μg/mL where ASCs had been shown to die due to chemical stress [10].

We hypothesized that:

(a) Gene expression of osteo-associated commitment would be enhanced with more aCaP nanoparticles in DMEM in a dose-dependent manner, while gene expression of endothelial cell commitment, chondrogenic and adipogenic commitment would be reduced in the presence of such nanoparticles.
(b) Gene expression changes would be more prominent at two weeks compared to 1 week.
(c) Human ASCs of three donors would behave individually different from each other with regard to gene expression changes—however, similar trends would occur.

## 2. Materials and Methods

### 2.1. Synthesis of aCaP Nanoparticles

**Figure 1:**
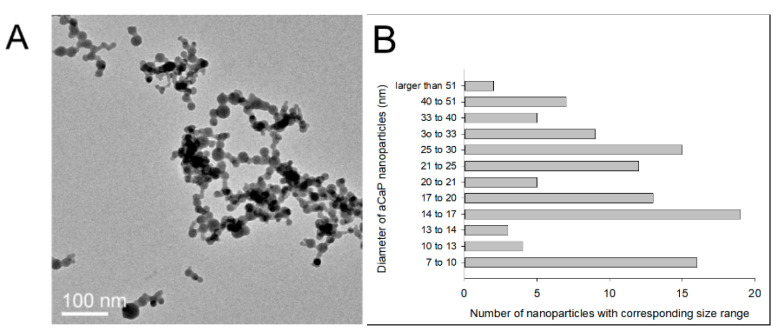
TEM image of the aCaP nanoparticles used in this study (**A**) and size distribution (**B**).

The aCaP nanoparticles (Ca/P = 1.5) were synthesized by flame spray pyrolysis according to Loher et al. [24] using calcium-2-ethylhexanoic salt, which was synthesized with calcium hydroxide (Riedel de Haen, Ph. Eur.) and ethylhexanoic (Sigma-Aldrich, St. Louis, MO, USA) and tributyl phosphate (Sigma-Aldrich, 98%). Transmission electron microscopy (TEM, FEI, Philips CM 12) was used to assess particle morphology and to measure the particles’ primary diameter (~22 nm, Figure 1).

### 2.2. Cell Isolation

Human ASCs were isolated from adipose tissue with the informed consent of the patient according to Swiss (Züricher Kantonale Ethik-Kommission KEK-ZH: StV 7-2009) and international ethical guidelines (ClinicalTrials.gov Identifier: NCT01218945) as reported earlier [25]. The extraction procedure was performed after Zuk et al. [13], and approved by the Swiss ethical guidelines (KEK-ZH: StV 7-2009). ASCs were characterized by established procedures [10,26]. Of the 30 isolated primary ASC lines [25], three donors were randomly selected: Donor D1: female, 45 years, subabdominal fat; donor D2: male, 39 years, subabdominal fat; donor D3: female, 41 years, abdominal fat.

### 2.3. Multilineage Cell Differentiation

Lineage-specific differentiation of ASCs towards the osteoblast, the adipogenic, the endothelial, and the chondrogenic cell lineage had been achieved previously [27], using cell culture media supplementation [13]. Osteogenic differentiation was evaluated by Von Kossa and Alizarin red staining, CD31 immunohistochemical staining was used to see the endothelial cell differentiation, and Alcian Blue staining to verify chondrogenesis. To visualize the adipogenic differentiation, Oil Red O staining was performed. For the primary ASCs used in this study, these four in vitro differentiations had been tested and performed before, and used according to induction media. The results were presented earlier [27,28].

### 2.4. ASC Cultivation with aCaP Nanoparticles

For the aCaP experiments and the controls, 200,000 of human ASCs of three donors (biological replicates *n* = 3) were cultivated in 6-well plates using 2 mL DMEM medium with 10% FBS and 50 µg mL^−1^ gentamycine for 1 or 2 weeks in a humidified atmosphere of 95% air and 5% CO_2_ at 37 °C. The medium was changed every 3 or 4 days, where the aCaP nanoparticles were replaced with every medium change. Passages used for the experiments were P5 (D1), P3 (D2), and P7 (D3). As for the medium spiked with aCaP nanoparticles, a suspension of 3 mg/mL aCaP nanoparticles in pure H_2_O was prepared and sonicated for 10 min in an ultrasound bath to get a final concentration of 5 µg/mL or 50 µg/mL aCaP; the stock solution was diluted with complete cell culture medium. Aliquots of 3 mL were taken from this suspension and added to the ASCs. Cells that were cultivated without nanoparticles served as a negative control. Furthermore, cells that were cultivated in an osteogenic induction medium served as a positive control. Induction medium consisted of DMEM with 10% FBS, 50 µg mL^−1^ gentamycine, 10 mM β-glycerophosphate, 50 µM ascorbic-2-phosphate and 100 nM dexamethasone. At the end of the experiments at 1 or 2 weeks, respectively, cells were collected for qPCR. The sample size was *n* = 3 (three experiments per donor), and technical replicates for qPCR were also *n* = 3.

### 2.5. Quantitative Real Time PCR

Total RNA was extracted from the ASCs using RNeasy Mini Kit (Qiagen, Hilden, Germany) according to the manufacturer’s instructions. The RNA was quantified using Nanodrop ND-1000 Spectrophotometer (Witec, Sursee, Switzerland), and 250 ng RNA was reversed transcribed into cDNA using oligo-dT primers (Invitrogen), dNTP mix (Invitrogen, Waltham, MA, USA), DTT (Invitrogen), 5x FSB (Invitrogen), RNA inhibitor (Applied Biosystem, Waltham, MA, USA), and Superscript III reverse transcriptase (Invitrogen). Quantitative PCR was performed using the SYBR^®^ Green master mix (Applied Biosystems), as well as primers synthesized by Microsynth (Balgach, Switzerland). For primer sequences, see Table S1.

Primers for mesenchymal stem cells CD73, CD90 and CD105, for CD31 and CD34 (markers of endothelial cells) [29], for Runx2 and ALP (early osteogenesis), for collagen I (medium osteogenesis) and osteocalcin (late osteogenesis), for PPAR-γ-2 (key transcription factor during adipogenesis) [30], and Sox9 (key transcription factor for chondrogenesis) [31] were used.

### 2.6. Measurement of Free Calcium and Phosphate Ion Concentrations

The calcium ions in the cell culture supernatant were determined by photometric analysis. Calcium ions and o-cresolphthalein form a chromogenic complex in which the calcium ion concentration is proportional to the measured absorbance at 575 nm. For this, the Calcium Colometric Assay Kit (Sigma-Aldrich) was used and performed according to the manufacturer’s instructions. All samples and standards were run in duplicates, and 50 µL samples were added per well. For the quantitative assessment of the phosphate ions in the supernatant, no pretreatment was required. The phosphate concentration was evaluated based on the color intensity measured at 620 nm, since malachite green dye and molybdate, together with inorganic phosphate, form a stable complex. Samples of 50 µL of the cell culture supernatant were analyzed with the QuantiChrom Phosphate Assay Kit (BioAssay Systems, Hayward, CA, USA) and according to the manufacturer’s instructions.

### 2.7. Statistics

The data were analyzed with StatView 5.0.1 software. One-way statistical analysis of variance (ANOVA) was conducted to test the significance of differences between different concentrations of aCaP nanoparticles and control. Unpaired t-tests were performed to compare time points, 7 and 14 days of incubation, respectively. Two-way ANOVA was conducted to test significant differences in phosphate and calcium ion concentrations, respectively, with levels of time and concentration. Pairwise comparison probabilities (*p*) were calculated using the Fisher’s PLSD post hoc test to evaluate differences between the groups. *p* values < 0.05 were considered significant (denoted as *); for *p* < 0.01 ** and for *p* < 0.001 ***. Values were expressed as means ± standard deviations.

## 3. Results

### 3.1. Impact of aCaP Nanoparticles on Gene Expression of Stem Cells

**Figure 2:**
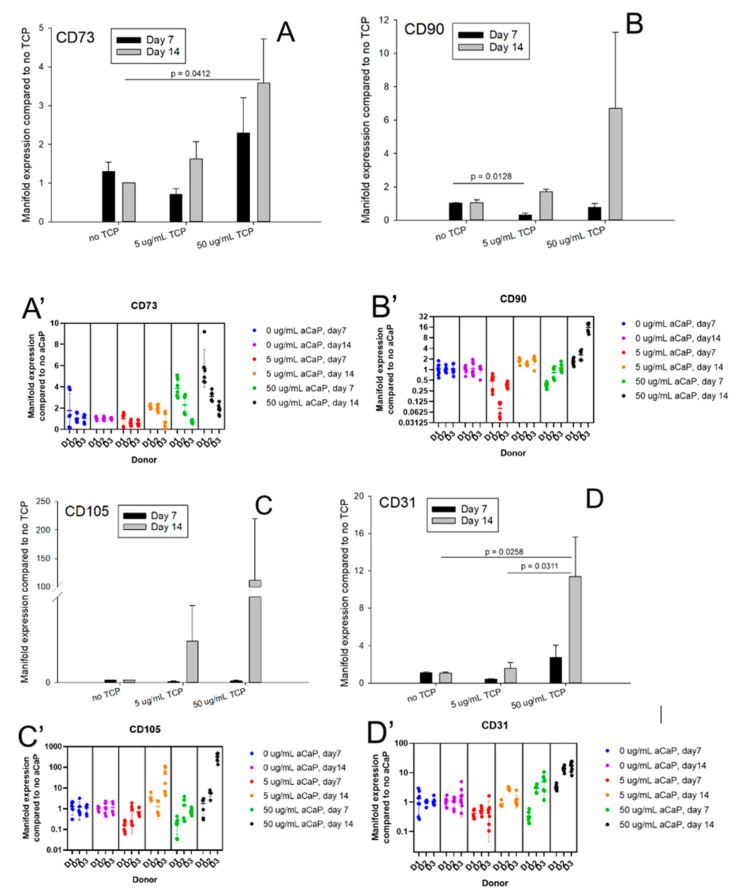

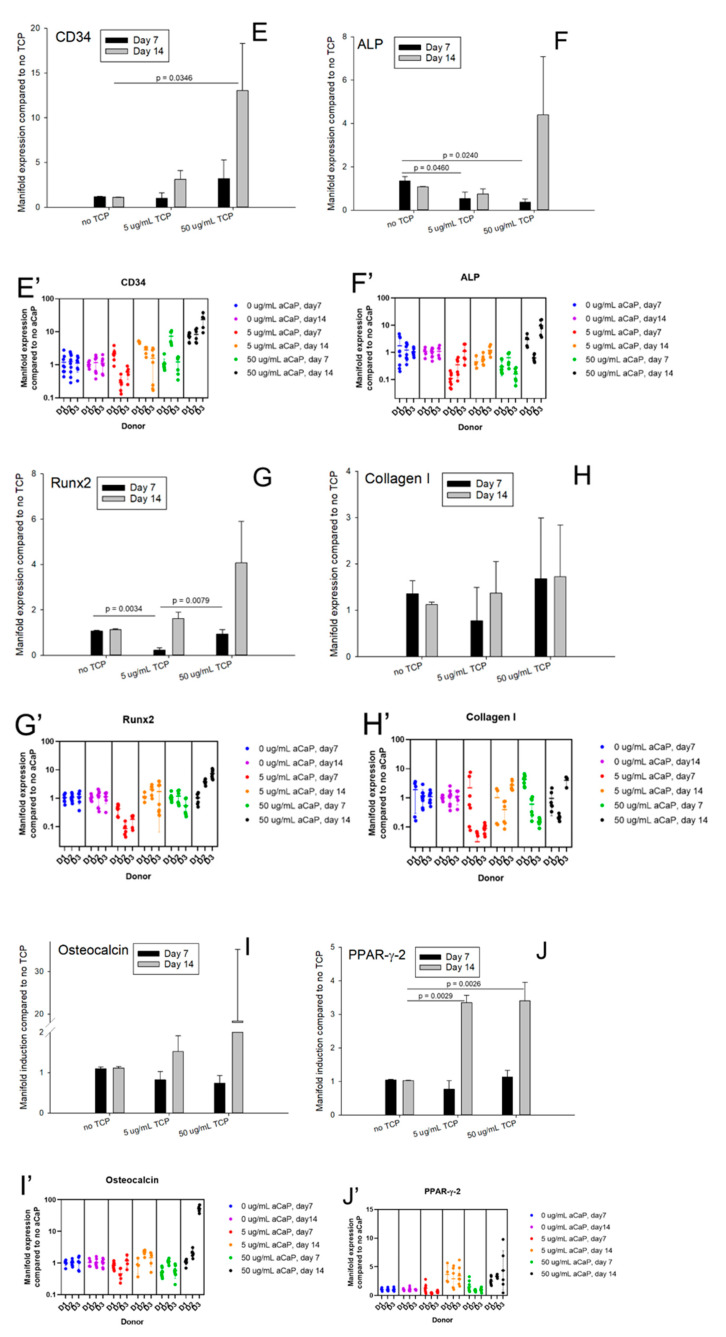

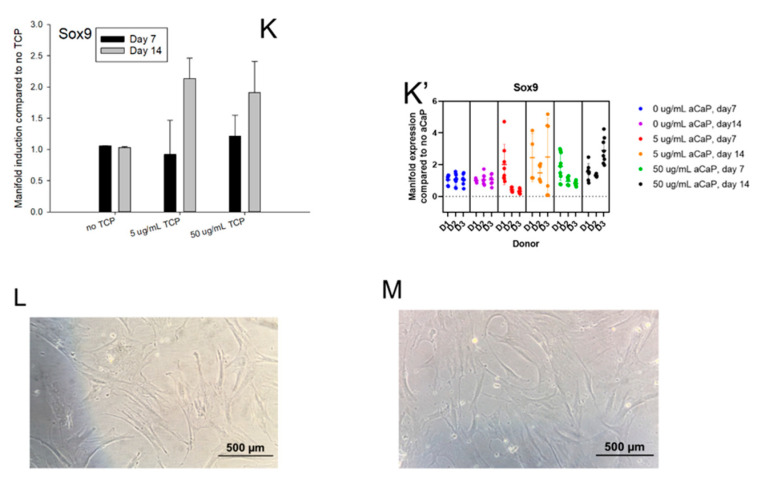
Average manifold induction of different genes averaged for three human donors (**A**–**K**) and corresponding nested plots for the three donors (D1, D2, and D2) with means and standard deviations for each donor (**A’**–**K’**). Representative images for 0 aCaP (**L**) and 50 µg/mL aCaP (**M**) after 14 days. Experiments were carried out for three conditions and two time points, with 0, 5, or 50 µg/mL aCaP nanoparticles (TCP = aCaP nanoparticles) and induction for 7 or 14 days in culture, respectively. Results are given as means, and error bars denote standard deviations. For gene expression in osteogenic culture medium, see Appendix A, Figure A1.

Gene expression of 11 target genes was assessed for two time points and for two concentrations of aCaP nanoparticles. Figure 2 shows the average manifold expression from three different donors compared to the basal culture medium without aCaP nanoparticles (=no TCP, no tricalciumphosphate). Stem cell markers were mainly unaffected, with two exceptions; one for CD73, where a significant upregulation was found for 50 µg/mL aCaP at day 14 compared to control (no aCaP). Moreover, the second for CD90, where it experienced a downregulation, when 5 µg/mL aCaP at day 7 were compared with the control.

Regarding the two endothelial cell markers, CD31 and CD34, respectively, gene expression averaged over the three donors showed a significant increase if the value at day 14 with 50 µg/mL aCaP was compared to the control (no TCP). ALP as a representative early osteogenic differentiation marker was downregulated at day 7 in a dose-dependent manner, while Runx2 was downregulated at day 7 when 5 µg/mL aCaP were compared to the control; however, it was upregulated when 50 µg/mL were compared to 5 µg/mL. Further osteogenic marker genes, such as collagen I and osteocalcin, were not affected. Finally, PPAR-γ-2, representing one important marker during adipogenesis, was upregulated at day 14 for both 5 and 50 µg/mL aCaP, when compared to the control, while Sox 9 (important for chondrogenesis) was not affected.

To get more insight into the individual response of each donor and to show individual comparisons at a glance (Figure 2A’–K’), we provide three tables (Table 1, Table 2 and Table 3). They denote changes determined when 5 µg/mL aCaP were compared to control (Table 1), when 50 µg/mL aCaP were compared with 5 µg/mL aCaP (Table 2) and when expression at day 14 was compared to day 7 (Table 3). Further quantitative information can be found in the Supplementary Figures S1–S6.

When screening the impact of 5 µg/mL aCaP nanoparticles suspended in basal culture medium on the gene expression of three different hASC donors, we found that after an incubation period of 1 week, several genes were significantly downregulated; in two out of three donors, Runx2 and collagen I were downregulated, as well as PPAR-γ-2 and Sox9 (Table 1). When comparing 5 µg/mL aCaP with particle-free conditions (control) at two weeks of incubation, two out of three donors showed no significant differences.

An increase of the aCaP nanoparticle concentration from 5 to 50 µg/mL suspended in DMEM revealed a significant upregulation of Sox9 gene expression in two out of three donors at 1 week of incubation, while osteocalcin experienced a downregulation. However, at two weeks, Sox9 expression did not show any significant changes anymore (in all three donors), while ALP and Runx2 revealed an upregulation in two out of three donors (Table 2). Moreover, CD31 was significantly upregulated, as well as the stem cell markers CD73 and CD105 (in two out of three donors) (Table 2). 

An obvious result for comparing the gene expression at two weeks with the time point at one week of incubation was a general upregulation of many genes considered here. Specifically, osteocalcin was significantly upregulated in all three donors for the 5 µg/mL experiment and in two out of three donors for the 50 µg/mL experiment, although the average of the three donors, gene expression was not showing any significance (Figure 2I). In addition, both endothelial cell marker genes and the stem cell markers CD73 and CD90 were upregulated at 5 µg/mL aCaP. As for the 50 µg/mL aCaP experiment, besides the osteocalcin also CD105, PPAR-γ-2, and Sox9 were significantly upregulated (Table 3). Individual profiling, thus, reveals the biological diversity and the different sensitivity of human ASCs, harvested from different donors, with respect to their changes in commitment when exposed to aCaP nanoparticles. 

### 3.2. Free Calcium and Phosphate Concentrations

**Figure 3:**
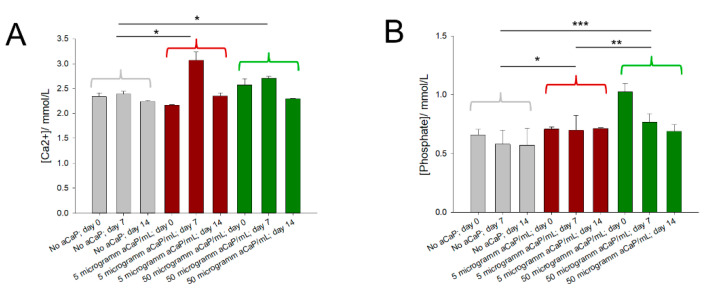
Average free calcium concentrations (**A**) and phosphate concentrations (**B**) for three conditions and two three points, with 0, 5, or 50 µg/mL aCaP nanoparticles and initially, as well as 7 or 14 days in culture, respectively. Two-way ANOVA for Ca^2+^ revealed significant differences between day 0 and day 7 (*p* = 0.0003), day 7 and day 14 (*p* = 0.0001), 0 and 5 µg/mL aCaP (*p* = 0.0113, marked with *), as well as 0 and 50 µg/mL aCaP (*p* = 0.0132, marked with *). Two-way ANOVA for phosphate revealed significant differences between day 0 and day 7 (*p* = 0.0323), day 0 and day 14 (*p* = 0.0027), 0 and 5 µg/mL aCaP (*p* = 0.0300, marked with *), 0 and 50 µg/mL aCaP (*p* < 0.0001, marked with ***), as well as 5 and 50 µg/mL aCaP (*p* = 0.0100, marked with **). Error bars indicate standard deviations.

Initially and at time points 7 and 14 days, the free Ca^2+^ ion concentration, as well as the phosphate P_i_ concentrations, were assessed in the culture media. The analysis revealed that calcium ion concentrations were higher than provided by the basal culture medium DMEM, where a 1.8 mM concentration is given. As for the phosphate concentration, it was lower compared to the concentration provided by DMEM, given with 0.9 mM (Figure 3). Measured Ca^2+^ concentrations were slightly fluctuating over time, with the highest values on day 7 for all three different concentrations of 0, 5, and 50 µg/mL aCaP, respectively (Figure 2A). Measured phosphate concentrations remained quite stable over time, but with different levels for each aCaP concentration. They were on average lowest for the medium without aCaP, as well as for medium with 5 µg/mL aCaP; and highest for medium with 50 µg/mL aCaP, respectively. The saturation status for all conditions was calculated (Table 4).

## 4. Discussion

Large bone defects demand accurate bone substitutes that support the healing process [33]. The gold standard in clinics is still using grafts from the iliac crest, sometimes mixed with a bone substitute like Bio-Oss^®^ [34]. However, such autologous bone grafts may have disadvantages like donor site morbidity besides limited availability. In addition, a second intervention is needed to provide the autologous graft for the defect. Finally, patient comorbidities and the possibility of infection must be considered. Hence, tissue engineering of bone grafts is one viable option to address those problems, arising from the clinical treatment of critical size bone defects [35].

During the last decades, a lot of research has been performed in terms of bone tissue engineering, where stem cells were used to support the regeneration and the formation of new bone at the defect site [36,37,38]. Specifically, the differentiation of stem cells towards the osteoblast phenotype has been under view [39]. Under laboratory conditions, the addition of supplements to the basal cell culture medium has been established [13,40]. Reliable and reproducible protocols for osteogenic differentiation have been determined and practiced. Moreover, stage-specific expression of osteogenic marker genes have been defined [39], and the impact of several supplements, such as dexamethasone [41], ascorbic acid, or β-glycerophosphate, on the osteogenic differentiation of stem cells have been tested [42].

Among the supplements for osteogenic differentiation, the external phosphate source is a key parameter. It should be provided in addition to the phosphate in the culture medium that usually contains 0.9 mM phosphate (Pi = all phosphate species; including PO_4_^3^^−^, HPO_4_^2^^−^, H_2_PO_4_^−^, H_3_PO_4_ depending on the pH). While β-glycerophosphate in a 10 mM concentration facilitates the mineralization of the extracellular matrix, other phosphate sources, such as a buffer of sodium dihydrogen phosphate with disodium hydrogenphosphate [18] or polyphosphate [20], can be used. Moreover, besides the standard osteogenic culture medium originally proposed by Zuk et al. [13], there are other osteogenic induction media at hand, with additional vitamin D_3_ and/or BMP-2 [19] or a bioactive glass component [43]. On the other hand, biomineralization demands calcium, where the continuous remodeling of bone releases Ca^2+^ ions, which may unite with phosphate to different calcium phosphate phases [32]. Among those phases, calcium-deficient hydroxyapatite (CDHAp) is very insoluble, with a p*K*_s0_ of 85 at 37 °C [32], while amorphous calcium phosphate is much better soluble. Notably, Ca^2+^ ions were reported to enhance and support osteogenic differentiation [44,45].

**Figure A1:**
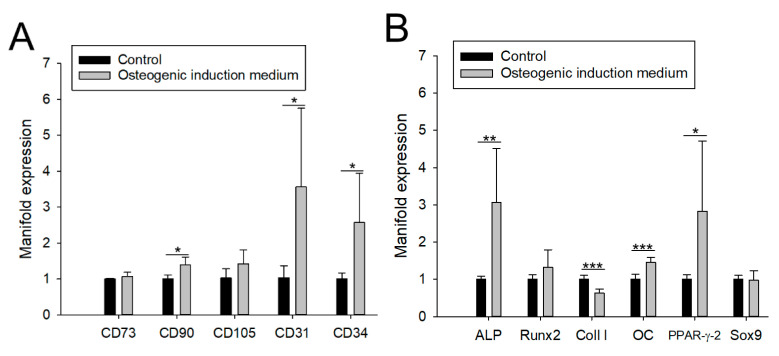
Average manifold induction of different genes. Experiments were carried out with osteogenic induction culture medium (see Section 2 for supplements) and 14 days in culture. Markers for stem cells, such as CD73, CD90 and CD105; and endothelial cells, such as CD31 and CD34, respectively (**A**) and markers for osteogenesis (ALP, Runx2, Collagen I and OC = osteocalcin), adipogenesis (PPAR-γ-2) and chondrogenesis (Sox9) (**B**) are shown. Results are given as means, and error bars denote standard deviations. Key: *p* < 0.05 (*); *p* < 0.01 (**) and *p* < 0.001 (***).

In this study, we addressed the effects of amorphous calcium phosphate nanoparticles suspended in basal culture medium DMEM without any further supplementation (no β-glycerophosphate, no additional phosphate buffer, nor polyphosphate). For this purpose, ASCs of three human donors were exposed to DMEM either with no aCaP nanoparticles (control) or with 5 or 50 µg/mL for one and two weeks, respectively. In addition, ASCs cultured in an osteogenic culture medium served as a positive control (Figure A1). As for the donors, they were all aged between 39 and 45 years, and they were patients in the Clinic for Plastic Surgery and Hand Surgery at the University Hospital Zurich, Switzerland, during 2010 and 2011 (KEK-ZH: StV 7-2009).

Different rationales stand behind this approach. First, suspended nanoparticles may trigger the osteogenic commitment without further supplementation as they provide a calcium and phosphate source—without being toxic [5]. Like this, an easy tool would be available for the change in stem cell commitment and for in vitro preparation of bone constructs. Second, the inter-donor comparison might be of interest in the discussion of individual sensitivity towards these nanoparticles. Third, calcium phosphate nanoparticles are used as delivery systems for RNA [46], and our study might elucidate the effects of the aCaP nanoparticles on the differentiation behavior of stem cells without RNA loading (aCaP nanoparticles as mere vehicles). 

Major findings were that the suspended aCaP nanoparticles enhanced gene expression of CD73, CD31, CD34, and PPAR-γ-2 for the 50 µg/mL concentration and a 2-week exposure, while they reduced gene expression of ALP and Runx2 for 5 µg/mL concentrations and a 1-week exposure (Figure 2A–K). Importantly, these results were obtained from the average gene expression of three human donors. A closer look at each individual donor, however, showed a non-negligible inter-donor variability (Figure 2A’–K’), stressing the need to consider individual cell responses towards such nanoparticle exposition. Finally, we assessed the free calcium and phosphate ion concentrations and found a generally increased calcium concentration compared with the theoretically given 1.8 mM (provided by the culture medium), while the phosphate concentration was in most cases lower than the theoretically 0.9 mM given by the medium.

The definition of minimal stem cell criteria by Dominici et al. includes the gene expression of CD73, CD90, and CD105 [17]. In our study, we determined a significantly increased expression of CD73 in the presence of 50 µg/mL aCaP nanoparticles after two weeks of exposure, averaged for the three donors. Recently, an ALP^+^/CD73^+^ subpopulation of human ASCs had been shown to exhibit an enhanced osteogenic potential compared with unsorted ASCs in conventional osteogenic culture medium [28]. Moreover, CD73 knock-out mice were reported to have a delayed bone regeneration and also a reduced bone matrix deposition. These findings were explained by CD73 as a key regulator of skeletal growth and an osteoblast activator. Thus, if osteogenic commitment is desired, increased expression of CD73 can be judged as a positive effect on the ASC commitment.

Gene expression of typical endothelial cell marker CD31 was also significantly increased in the presence of aCaP nanoparticles after a 2-week exposure, supported by significantly higher CD34 gene expression [47]. It is well known that induced pluripotent stem cells (iPSCs) [48], and ASCs are capable of endothelial cell differentiation, although induction by endothelial cell differentiation medium EGM-2MV showed differences in species, with rat ASCs differentiating more easily towards endothelial cells than human ASCs [49]. During bone healing, angiogenesis is a crucial factor, and many bone tissue engineering approaches; therefore, not only focus on the osteogenic potential, but also on the angiogenic potential [50]. For example, this osteo/angiogenic potential was addressed by a combination of poly lactic-co-glycolic acid incorporated with micro-nano bioactive glass and mouse bone mesenchymal stem cells [51] or β-tricalcium phosphate seeded with canine bone marrow mesenchymal stem cells [52]. Therefore, our findings of an increase in endothelial cell marker gene expression are beneficial, even if it is only a slight change in commitment towards the endothelial phenotype, most probably undergone in a subpopulation of the otherwise heterogeneous ASC culture [28]. Interestingly, we found a very similar and significant increase in CD31 and CD34 gene expression in conventional osteogenic induction culture medium for a 2-week experiment (Figure A1), supporting the usefulness of aCaP nanoparticles to act as an alternative for osteogenic supplementation. Besides, the significant induction of CD34 gene expression in ASCs might also be attributed to replicative capacity in addition to endothelial cell-related pathways [53].

The delicate balance of ASCs towards an osteogenic or an adipogenic commitment is well known [54]. In general, osteogenesis and adipogenesis are mutually exclusive or in inverse relationships. For the average of the three donors, we found a significantly increased PPAR-γ-2 expression (Figure 2J), a central marker during adipogenesis. The same was found for a 2-week experiment a normal osteogenic induction medium (Figure A1,B) [30]. In contrast, ALP expression (early osteogenic marker) was significantly downregulated at 1 week in the presence of 5 µg/mL aCaP, while Runx2 (a key regulator for osteogenesis [55]) was downregulated for 5, but upregulated for 50 µg/mL aCaP during the same time period. However, a look at individual responses towards aCaP exposition revealed that 2/3 donors showed an individual downregulation of PPAR-γ-2 at 1 week (5 µg/mL compared to no aCaP) and only one out of three donors a significant upregulation at 1 week (50 µg/mL compared to 5 µg/mL aCaP).

As for ALP, individual consideration of the gene expression dynamics revealed a downregulation in 1/3 during the first seven days, followed by a significant upregulation in the following seven days by 2/3 of the donors (50 µg/mL compared to 5 µg/mL aCaP), going along with the determined upregulation of ALP under normal osteogenic culture medium conditions (Figure 1A,B). The commitment of ASCs exposed to conventional osteogenic induction medium at two weeks with a simultaneous upregulation of PPAR-γ-2 and ALP (both around 3-fold), which is similar to our aCaP results, may again reflect different subpopulations in the ASCs of the three donors; with different amounts of “osteo-prone” and “adipo-prone” cells [28].

It seems that the sensitive balance favoring either adipo- or osteogenesis with its reported key factor *Msx2*, regulating this switch, and its downstream transcription factor Runx2 might be dependent on the specific composition of the subpopulations given in the isolated ASCs [39]. Otherwise, the individual dynamics would not differ to such an extent as found here. It has been shown before that ASCs from each individual donor may vary quite a lot with respect to multilineage differentiation capacity, although they were isolated by the same protocol [56].

Besides the individual response of the three donors towards the aCaP suspension, the complex kinetics of aCaP transformation to HAp [8], all thermodynamic equilibria of calcium phosphate phases with dissolution and precipitation [7,12,57] also have to be accounted for the response of the (heterogeneous) ASCs to aCaP nanoparticle exposition.

In this regard, some worthwhile considerations about the calcium phosphate phases must be made that could be present in our experimental system besides the initially suspended aCaP nanoparticles. The free Ca^2+^ and the P_i_ ion concentrations were assessed in the culture media (Figure 3). Although our DMEM basal culture medium should have had a total calcium ion concentration of 1.8 mM, we measured around 2.3 mM in all samples where no aCaP was suspended (control). Obviously, a higher basal Ca^2+^ concentration was present. For the samples with additional aCaP nanoparticles, an increase in Ca^2+^ on top of the basal concentration was measured that peaked at day 7 and decreased a bit on day 14. On the other hand, P_i_ concentrations were overall lower than theoretically given by the DMEM medium (0.9 mM); they were around 0.6 mM without further aCaP addition, as well as roughly 0.7 mM (at 5 µg/mL aCaP) and 0.8 mM (at 50 µg/mL aCaP), respectively.

Based on those concentrations, we calculated the reaction coefficient *Q*_s0_ (ionic activity product based on actual concentrations, no correction for ionic strength) and compared them with solubility constants (*K*_s0_) reported and determined at 37 °C for a set of different calcium phosphate phases [32] (Table 4). First, we considered hydroxyapatite (HAp) formation because the transformation of aCaP into HAp is a well-known reaction [8], and found that all our systems were oversaturated with regard to HAp (for all systems log*Q_s0_*-log*K_s0_* > 70). Obviously, the calcium and phosphate concentrations were metastable with respect to HAp precipitation.

The examination of further phases revealed that the systems were also oversaturated with respect to octocalciumphophate, but undersaturated with respect to monocalciumphosphate monohydrate (for all systems log*Q_s0_*-log*K_s0_* < −7). From the actual concentrations of the calcium ions and a comparison to their theoretical concentration under the assumption that all aCaP nanoparticles would have been dissolved, it was concluded that some of the aCaP had dissolved, but not all; because actual concentrations were still lower at 50 µg/mL aCaP where a theoretical [Ca^2+^] increase of 26.8% was maximally expected (from 2.3 mM (baseline here) to approximately 2.9 mM (if all aCaP had dissolved)), with a measured increase to around 2.5 mM only. Moreover, the saturation status with respect to further calcium phosphate phases is summarized in Table 4. Our systems were oversaturated with respect to α-TCP, β-TCP, CDHAp, tetracalciumphosphate, besides the already mentioned HAp and octacalciumphosphate. For two of the considered calcium phosphate phases, the saturation status was near equilibrium, for dicalciumphosphate dehydrate (brushite) and dicalciumphosphate anhydrate (monetite), respectively. Overall, the chemical composition for all conditions tested here remained approximately constant over time.

Having this chemical composition in mind, with only small changes in calcium and phosphate ion concentrations because of aCaP nanoparticle suspension, the high impact on the gene expression of human ASCs gains particular attention. It has to be stressed that without changing the saturation status of the culture medium much, aCaP nanoparticles impact ASC gene expression quite impressively. Therefore, we mainly conclude that there is a high sensitivity of human ASCs towards aCaP nanoparticles suspended in the culture medium at very low concentrations, such as 5 or 50 µg/mL. Although free ion concentrations did not vary to a great extent, the cells sensed the suspended aCaP nanoparticles and possibly their transformation products, such as HAp, either by adherence of the particles on their cell surfaces or also by incorporation of those nanoparticles. Like this, aCaP nanoparticles might be used in the future as a suitable phosphate source in such small concentrations as applied here. Further, they could be used to trigger a desired osteogenic and angiogenic commitment in mesenchymal stem cells—particularly in combination with biomaterials for bone tissue engineering.

### Limitations

As we addressed only short term commitment of human ASCs within a time frame of two weeks, the study is limited to this period. Longer experiments aiming to study full differentiation, not just changes in commitment, might be interesting and widen the perspective. A further limitation of the study is that we do not know how fast aCaP nanoparticles suspended in DMEM transform to HAp and how fast they dissolve. Furthermore, we cannot give information about agglomerations of aCaP nanoparticles in the culture medium, which might also affect the commitment of the stem cells.

## 5. Conclusions

The addition of aCaP nanoparticles to the basal culture medium as a suspension enhanced CD73, CD31, and CD34 gene expression of human ASCs, averaged over three donors. Osteogenic marker genes, such as ALP or Runx2, experienced a downregulation. However, individual responses revealed a high inter-donor variability, with ALP and Runx2 enhancements for distinct conditions: Two out of three donors exhibited a significant upregulation at day 14 and 50 µg/mL aCaP compared to 5 µg/mL aCaP.

Free calcium and phosphate ion concentrations showed an oversaturated status with respect to several calcium phosphate phases, among them hydroxyapatite. This metastable status, however, did not change much over the period of two weeks and for the concentrations of 5 or 50 µg/mL aCaP, respectively. We conclude that not only the free ion concentration changes affect the gene expression, but also the direct interaction of the suspended particles, either by cell surface adherence or by incorporation. Like this, the suspension of low concentrations of aCaP nanoparticles in normal culture medium DMEM may be used to tune stem cells towards an angiogenic/osteogenic commitment, which might be interesting in future bone tissue engineering approaches.

## Figures and Tables

**Table 1:** Changes in gene expression of ASCs from three Donors (D1–D3) in the presence of 5 µg/mL aCaP nanoparticles suspended in basal medium DMEM compared to basal medium DMEM without nanoparticles, after incubation of 7 days and 14 days, respectively. Detailed results are given in the Supporting Information Figures S1–S3. Key: ↑ = upregulation; ↓ = downregulation; * = *p* < 0.05; ** = *p* < 0.01; *** = *p* < 0.001; - = no significant change in gene expression. For example, ↑ * means upregulation at a significance level with *p* < 0.05.

	D1	D2	D3	D1	D2	D3
	from 0 to 5 µg/mL			from 0 to 5 µg/mL		
	Day 7			Day 14		
**CD73**	-	-	↓ **	-	-	-
**CD90**	-	-	↓ ***	↑ **	-	-
**CD105**	-	-	-	-	-	-
**CD31**	-	-	-	-	-	-
**CD34**	↑ *	-	-	-	-	-
**ALP**	-	-	-	-	↓ *	-
**Runx2**	↓ **	-	↓ **	-	-	-
**Collagen I**	-	↓ **	↓ ***	-	↓ *	-
**Osteocalcin**	-	↓ ***	-	-	↑ ***	-
**PPAR-γ-2**	-	↓ ***	↓ *	-	-	-
**Sox9**	-	↓ **	↓ ***	-	-	-

**Table 2:** Changes in gene expression of ASCs from three Donors (D1–D3) in the presence of 50 µg/mL aCaP. nanoparticles suspended in basal medium DMEM compared to 5 µg/mL aCaP nanoparticles suspended in basal medium DMEM, after incubation of 7 days and 14 days, respectively. Detailed results are given in the Figures S1–S3. Key: ↑ = upregulation; ↓ = downregulation; * = *p* < 0.05; ** = *p* < 0.01; *** = *p* < 0.001; - = no significant change in gene expression.

	D1	D2	D3	D1	D2	D3
	from 5 to 50 µg/mL			from 5 to 50 µg/mL		
	Day 7			Day 14		
**CD73**	-	-	-	↑ **	-	↑ **
**CD90**	-	-	↑ ***	-	-	↑ ***
**CD105**	-	-	-	-	↑ ***	↑ ***
**CD31**	-	-	↑ **	↑ ***	-	↑ ***
**CD34**	↓ **	-	-	-	-	↑ ***
**ALP**	-	-	↓ **	↑ **	-	↑ ***
**Runx2**	↑ ***	-	-	-	↑ **	↑ ***
**Collagen I**	-	-	-	-	-	-
**Osteocalcin**	↓ **	↑ ***	↓ *	-	-	↑ ***
**PPAR-γ-2**	-	↑ **	-	-	-	-
**Sox9**	-	↑ *	↑ **	-	-	-

**Table 3:** Changes in gene expression of ASCs from three Donors (D1–D3) at 2 weeks of incubation compared to 1 week in the presence of 5 µg/mL aCaP nanoparticles suspended in basal medium DMEM and 50 µg/mL aCaP nanoparticles suspended in basal medium DMEM, respectively. Detailed results are given in the Figures S4–S6. Key: ↑ = upregulation; ↓= downregulation; * = *p* < 0.05; ** = *p* < 0.01; ***= *p* < 0.001; - = no significant change in gene expression.

	D1	D2	D3	D1	D2	D3
	from 1 to 2 Weeks			from 1 to 2 Weeks		
	at 5 µg/mL aCaP			at 50 µg/mL a CaP		
**CD73**	↑ ***	↑ ***	-	-	-	↑ **
**CD90**	-	↑ ***	↑ ***	-	-	↑ ***
**CD105**	-	-	-	-	↑ *	↑ ***
**CD31**	-	↑ ***	↑ ***	-	-	↑ ***
**CD34**	↑ ***	-	↑ *	-	-	↑ ***
**ALP**	↑ **	-	-	-	-	↑ **
**Runx2**	↑ *	-	-	↓ **	-	↑ ***
**Collagen I**	-	-	↑ ***	-	-	↑ ***
**Osteocalcin**	↑ ***	↑ ***	↑ *	↑ ***	-	↑ ***
**PPAR-γ-2**	-	-	↑ ***	-	↑ ***	↑ *
**Sox9**	-	-	↑ **	↑ *	-	↑ ***

**Table 4:** Ionic activity products (*Qs0*) and differences between *logQs0-logKs0* for nine different conditions (numbered 1–9), i.e., no aCaP, 5, or 50 µg/mL aCaP and time points 0, 7, and 14 days, respectively, calculated based on measured free calcium and phosphate ion concentrations (see Figure 2). No corrections regarding ionic strength were made.

**A**							
		**DAY 0**		**DAY 7**		**DAY 14**	
**No aCaP**	[32]	1	1	2	2	3	3
	Solubility constant at 37 °C	ionic activity product		ionic activity product		ionic activity product	
Phase	*Ks0*	*Qs0*	*logQs0-logKs0*	*Qs0*	*logQs0-logKs0*	*Qs0*	*logQs0-logKs0*
Ca(H_2_PO_4_)_2_ H_2_O	0.072443596	1.014 × 10^−9^	−7.85	8.011 × 10^−10^	−7.96	7.282 × 10^−10^	−8.00
CaHPO_4_ 2H_2_O	2.34423 × 10^−7^	1.540 × 10^−6^	0.82	1.384 × 10^−6^	0.77	1.278 × 10^−6^	0.74
CaHPO_4_	9.54993 × 10^−8^	1.540 × 10^−6^	1.21	1.384 × 10^−6^	1.16	1.278 × 10^−6^	1.13
Ca_8_(HPO_4_)_2_(PO_4_)_4_ 5H_2_O	1.25893 × 10^−96^	7.285 × 10^−41^	55.76	4.029 × 10^−41^	55.51	2.187 × 10^−41^	55.24
alpha-Ca_3_(PO_4_)_2_	3.16228 × 10^−26^	5.543 × 10^−15^	11.24	4.585 × 10^−15^	11.16	3.660 × 10^−15^	11.06
beta-Ca_3_(PO_4_) _2_	3.16228 × 10^−30^	5.543 × 10^−15^	15.24	4.585 × 10^−15^	15.16	3.660 × 10^−15^	15.06
Ca_10-x_(HPO_4_)_x_(PO4)_6x_(OH)_2-x_ (0<x<1)	7.94328 × 10^−86^	1.703 × 10^−43^	42.33	9.640 × 10^−44^	42.08	4.902 × 10^−44^	41.79
Ca_10_(PO_4_)_6_(OH)_2_	6.3096 × 10^−118^	3.983 × 10^−46^	71.80	2.306 × 10^−46^	71.56	1.099 × 10^−46^	71.24
Ca_4_(PO4)_2_O	3.16228 × 10^−40^	1.296 × 10^−17^	22.61	1.097 × 10^−17^	22.54	8.205 × 10^−18^	22.41
**B**							
		**DAY 0**		**DAY 7**		**DAY 14**	
**5 uM aCaP**	[32]	4	4	5	5	6	6
	Solubility constant at 37 °C	ionic activity product		ionic activity product		ionic activity product	
Phase	*Ks0*	*Qs0*	*logQs0-logKs0*	*Qs0*	*logQs0-logKs0*	*Qs0*	*logQs0-logKs0*
Ca(H_2_PO_4_)_2_ H_2_O	0.072443596	1.091 × 10^−9^	−7.82	1.495 × 10^−9^	−7.69	1.199 × 10^−9^	−7.78
CaHPO_4_ 2H_2_O	2.34423 × 10^−7^	1.539 × 10^−6^	0.82	2.140 × 10^−6^	0.96	1.679 × 10^−6^	0.86
CaHPO_4_	9.54993 × 10^−8^	1.539 × 10^−6^	1.21	2.140 × 10^−6^	1.35	1.679 × 10^−6^	1.25
Ca_8_(HPO_4_)_2_(PO_4_)_4_ 5H_2_O	1.25893 × 10^−96^	6.255 × 10^−41^	55.70	9.013 × 10^−40^	56.85	1.240 × 10^−40^	55.99
alpha-Ca_3_(PO_4_)_2_	3.16228 × 10^−26^	5.140 × 10^−15^	11.21	1.403 × 10^−14^	11.65	6.632 × 10^−15^	11.32
beta-Ca_3_(PO_4_) _2_	3.16228 × 10^−30^	5.140 × 10^−15^	15.21	1.403 × 10^−14^	15.65	6.632 × 10^−15^	15.32
Ca_10-x_(HPO_4_)_x_(PO4)_6x_(OH)_2-x_ (0<x<1)	7.94328 × 10^−86^	1.358 × 10^−43^	42.23	2.761 × 10^−42^	43.54	2.917 × 10^−43^	42.56
Ca_10_(PO_4_)_6_(OH)_2_	6.3096 × 10^−118^	2.948 × 10^−46^	71.67	8.459 × 10^−45^	73.13	6.862 × 10^−46^	72.04
Ca_4_(PO4)_2_O	3.16228 × 10^−40^	1.116 × 10^−17^	22.55	4.298 × 10^−17^	23.13	1.560 × 10^−17^	22.69
**C**							
		**DAY 0**		**DAY 7**		**DAY 14**	
**50 uM aCaP**	[32]	7	7	8	8	9	9
	Solubility constant at 37 °C	ionic activity product		ionic activity product		ionic activity product	
Phase	*Ks0*	*Qs0*	*logQs0-logKs0*	*Qs0*	*logQs0-logKs0*	*Qs0*	*logQs0-logKs0*
Ca(H_2_PO_4_)_2_ H_2_O	0.072443596	2.707 × 10^−9^	−7.43	1.593 × 10^−9^	−7.66	1.094 × 10^−9^	−7.82
CaHPO_4_ 2H_2_O	2.34423 × 10^−7^	2.638 × 10^−6^	1.05	2.075 × 10^−6^	0.95	1.584 × 10^−6^	0.83
CaHPO_4_	9.54993 × 10^−8^	2.638 × 10^−6^	1.44	2.075 × 10^−6^	1.34	1.584 × 10^−6^	1.22
Ca_8_(HPO_4_)_2_(PO_4_)_4_ 5H_2_O	1.25893 × 10^−96^	2.224 × 10^−39^	57.25	5.848 × 10^−40^	56.67	8.306 × 10^−41^	55.82
alpha-Ca_3_(PO_4_)_2_	3.16228 × 10^−26^	1.788 × 10^−14^	11.75	1.165 × 10^−14^	11.57	5.754 × 10^−15^	11.26
beta-Ca_3_(PO_4_) _2_	3.16228 × 10^−30^	1.788 × 10^−14^	15.75	1.165 × 10^−14^	15.57	5.754 × 10^−15^	15.26
Ca_10-x_(HPO_4_)_x_(PO4)_6x_(OH)_2-x_ (0<x<1)	7.94328 × 10^−86^	5.715 × 10^−42^	43.86	1.582 × 10^−42^	43.30	1.905 × 10^−43^	42.38
Ca_10_(PO_4_)_6_(OH)_2_	6.3096 × 10^−118^	1.469 × 10^−44^	73.37	4.278 × 10^−45^	72.83	4.369 × 10^−46^	71.84
Ca_4_(PO4)_2_O	3.16228 × 10^−40^	4.595 × 10^−17^	23.16	3.151 × 10^−17^	23.00	1.320 × 10^−17^	22.62

## Data Availability

Not applicable.

## Supplementary Materials

The following are available online at https://www.mdpi.com/article/10.3390/biology10070675/s1, Figure S1: Average manifold induction of different genes for donor 1 (internal abbreviation F4). Experiments were carried out for three conditions and two time points, with 0, 5 or 50 µg/mL aCaP nanoparticles, denoted as Conc. = concentration and incubation for 7 or 14 days in culture, respectively, Figure S2: Average manifold induction of different genes for donor 2 (internal abbreviation F19). Experiments were carried out for three conditions and two time points, with 0, 5 or 50 µg/mL aCaP nanoparticles, denoted as Conc. = concentration and incubation for 7 or 14 days in culture, respectively, Figure S3: Average manifold induction of different genes for donor 3 (internal abbreviation F15). Experiments were carried out for three conditions and two time points, with 0, 5 or 50 µg/mL aCaP nanoparticles, denoted as Conc. = concentration and incubation for 7 or 14 days in culture, respectively, Figure S4: Average manifold induction of different genes for donor 1 (internal abbreviation F4). Experiments were carried out for three conditions and two time points, with 0, 5 or 50 µg/mL aCaP nanoparticles and at 7 or 14 days in culture, respectively. Key: TCP = amorphous calcium phosphate nanoparticles, Figure S5: Average manifold induction of different genes for donor 2 (internal abbreviation F19). Experiments were carried out for three conditions and two time points, with 0, 5 or 50 µg/mL aCaP nanoparticles and at 7 or 14 days in culture, respectively. Key: TCP = amorphous calcium phosphate nanoparticles, Figure S6: Average manifold induction of different genes for donor 3 (internal abbreviation F15). Experiments were carried out for three conditions and two time points, with 0, 5 or 50 µg/mL aCaP nanoparticles and at 7 or 14 days in culture, respectively. Key: TCP = amorphous calcium phosphate nanoparticles, Table S1: The sequences of forward and reverse primers

## Appendix A

Besides gene expression analysis after aCaP nanoparticle exposure, we have also performed a 2-week experiment where ASCs were exposed to an osteogenic induction medium. Gene expression of the same markers as determined in the aCaP nanoparticle experiments was analyzed (Figure A1).
