# Supplementary material for: Suspension of Amorphous Calcium Phosphate Nanoparticles Impact Commitment of Human Adipose-Derived Stem Cells In Vitro
Source: Biology (Basel). 2021 Jul 16;10(7):675. doi: 10.3390/biology10070675 (PMC8301486; doi:10.3390/biology10070675)
Supplement: Supplementary file 1 [file biology-10-00675-s001.zip › biology-1278324-supplementary.pdf]

Supporting Information

Suspension of amorphous calcium phosphate nanoparticles impact commitment of human adipose-derived stem cells *in vitro*

Petra Wolint[1], Lukas Näf[1], Désirée Schibler[1], Nora Hild[2], Wendelin J. Stark[2], Pietro Giovanoli[1], Maurizio Calcagni[1] and Johanna Buschmann[1,*]

[1]Division of Plastic and Hand Surgery, University Hospital Zurich, Zurich, 8091, Switzerland
[2]Institute for Chemical and Bioengineering, Department of Chemistry and Applied Biosciences, ETH Zurich, Zurich, 8093, Switzerland

[*]Corresponding author: johanna.buschmann@usz.ch

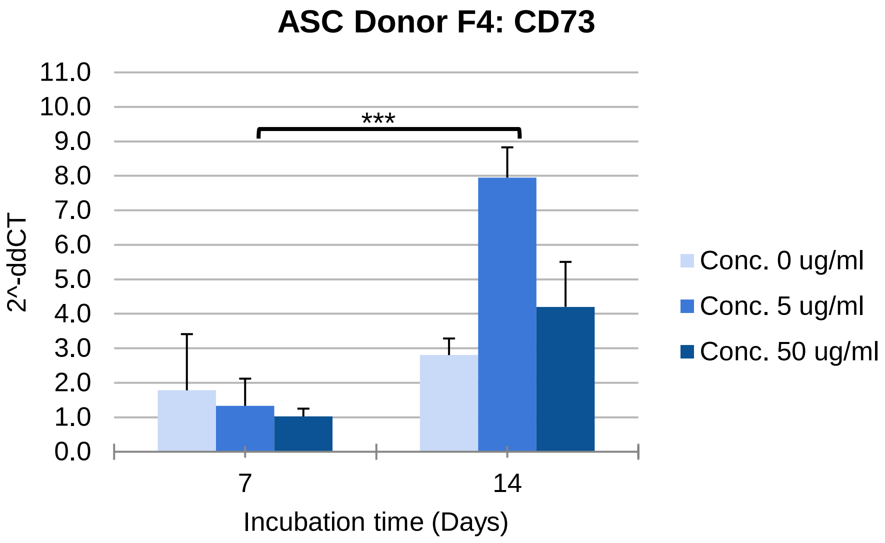
ASC Donor F4: CD73
11.0
10.0
9.0
8.0
7.0
6.0
5.0
4.0
3.0
2.0
1.0
0.0
2^-ddCT
***
7
14
Incubation time (Days)
Conc. 0 ug/ml
Conc. 5 ug/ml
Conc. 50 ug/ml

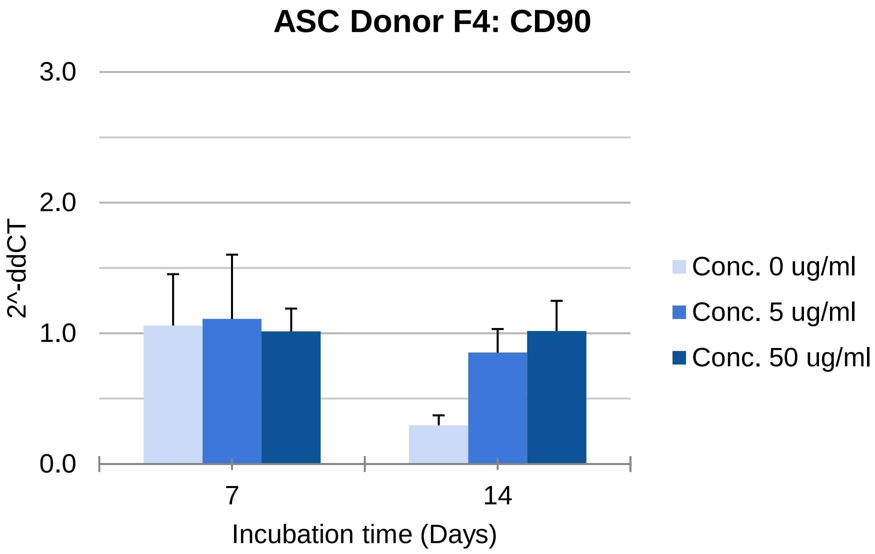
ASC Donor F4: CD90
3.0
2.0
1.0
0.0
2^-ddCT
7
14
Incubation time (Days)
Conc. 0 ug/ml
Conc. 5 ug/ml
Conc. 50 ug/ml

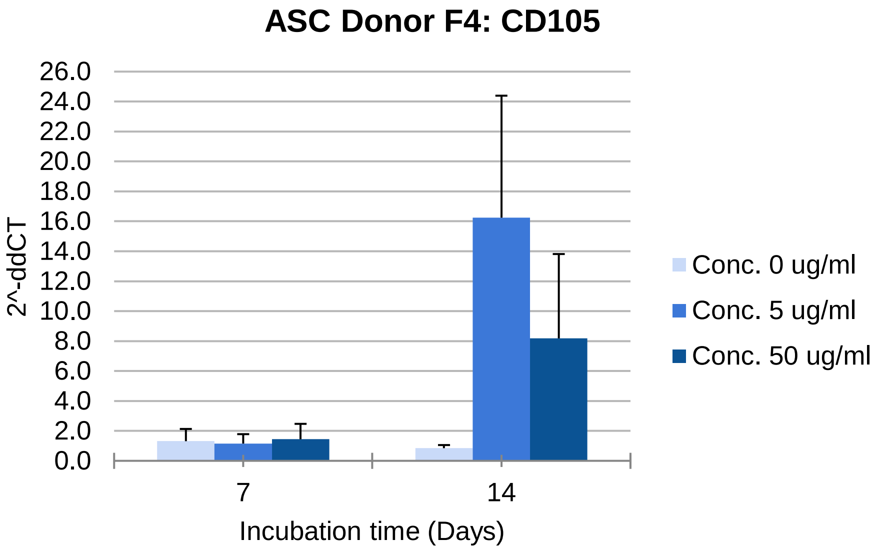
ASC Donor F4: CD105
26.0
24.0
22.0
20.0
18.0
16.0
14.0
12.0
10.0
8.0
6.0
4.0
2.0
0.0
2^-ddCT
7
14
Incubation time (Days)
Conc. 0 ug/ml
Conc. 5 ug/ml
Conc. 50 ug/ml

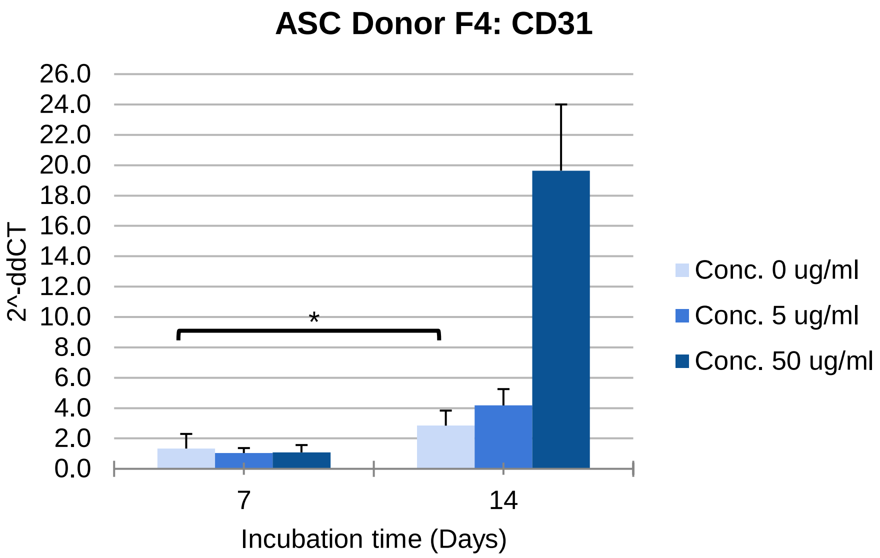
ASC Donor F4: CD31
26.0
24.0
22.0
20.0
18.0
16.0
14.0
12.0
10.0
8.0
6.0
4.0
2.0
0.0
2^-ddCT
*
7
14
Incubation time (Days)
Conc. 0 ug/ml
Conc. 5 ug/ml
Conc. 50 ug/ml

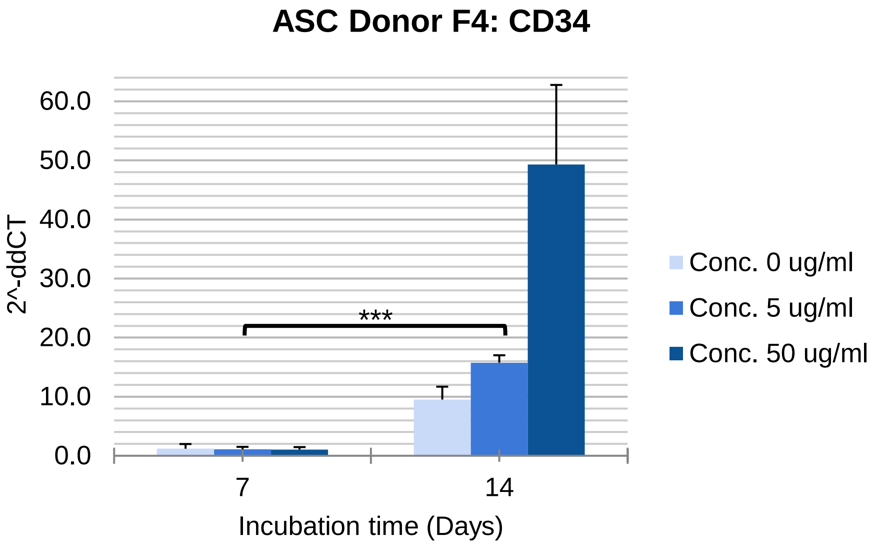
ASC Donor F4: CD34
60.0
50.0
40.0
30.0
20.0
10.0
0.0
2^-ddCT
***
7
14
Incubation time (Days)
Conc. 0 ug/ml
Conc. 5 ug/ml
Conc. 50 ug/ml

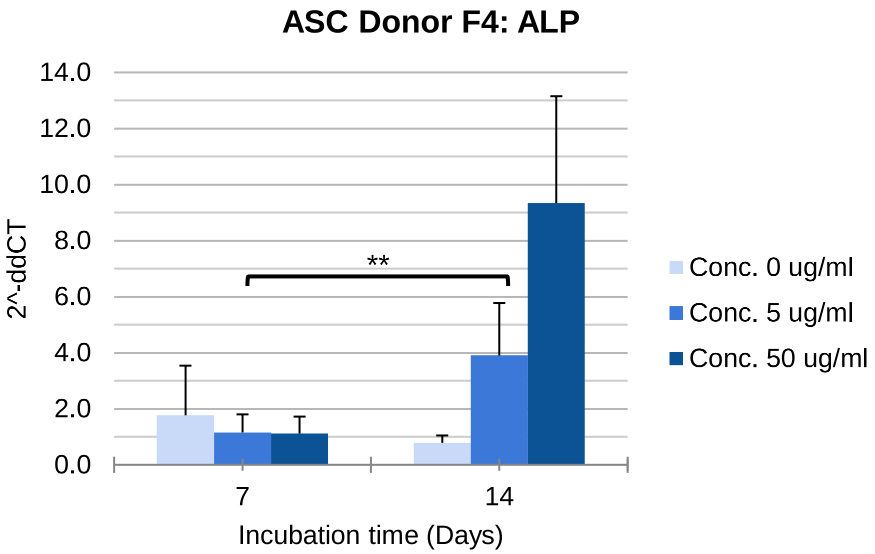
ASC Donor F4: ALP
14.0
12.0
10.0
8.0
6.0
4.0
2.0
0.0
2^-ddCT
**
7
14
Incubation time (Days)
Conc. 0 ug/ml
Conc. 5 ug/ml
Conc. 50 ug/ml

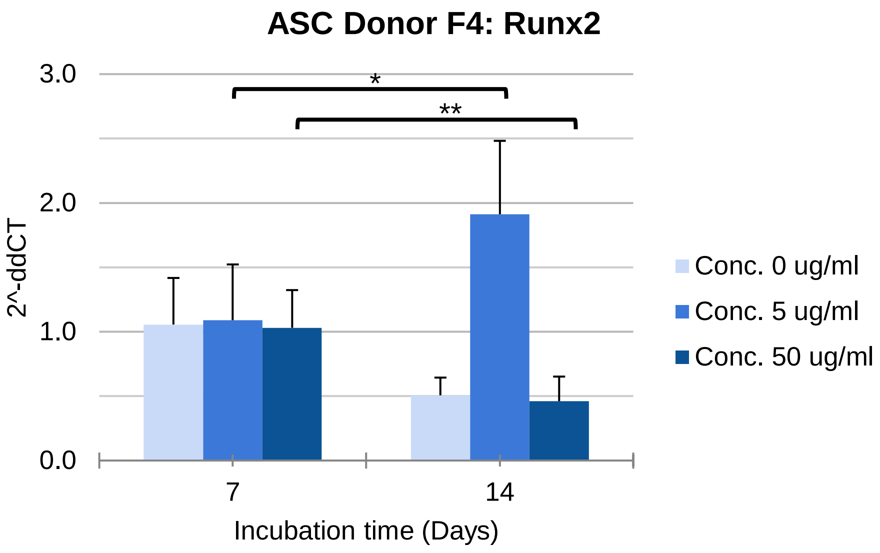
ASC Donor F4: Runx2
3.0
2.0
1.0
0.0
2^-ddCT
*
**
7
14
Incubation time (Days)
Conc. 0 ug/ml
Conc. 5 ug/ml
Conc. 50 ug/ml

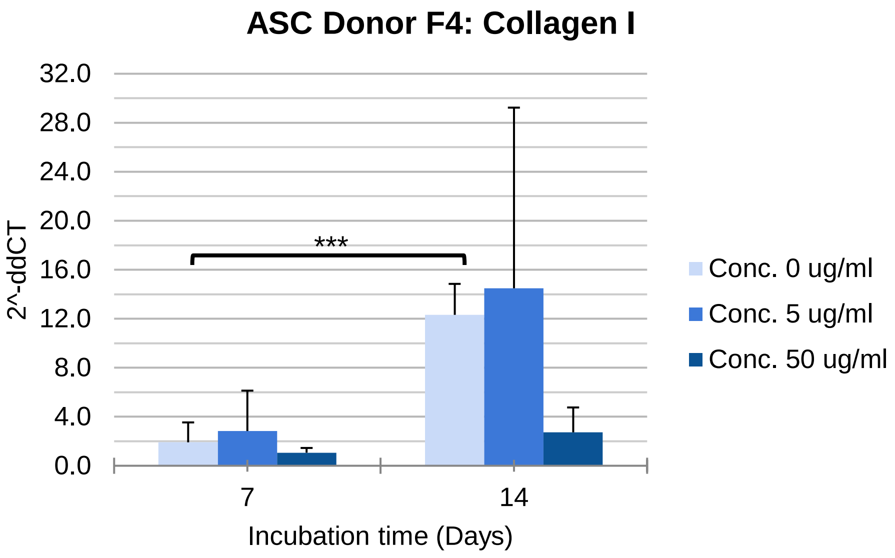
ASC Donor F4: Collagen I
32.0
28.0
24.0
20.0
16.0
12.0
8.0
4.0
0.0
2^-ddCT
***
7
14
Incubation time (Days)
Conc. 0 ug/ml
Conc. 5 ug/ml
Conc. 50 ug/ml

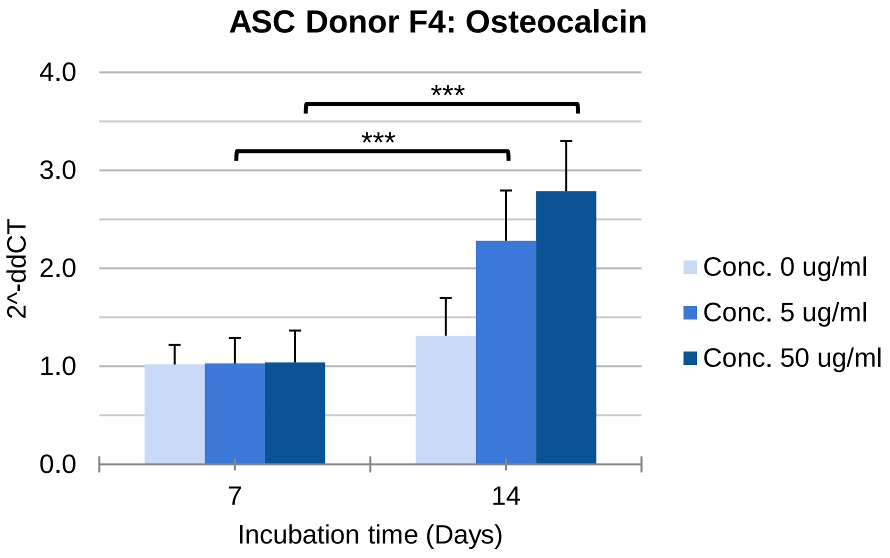
ASC Donor F4: Osteocalcin
4.0
3.0
2.0
1.0
0.0
2^-ddCT
***
***
7
14
Incubation time (Days)
Conc. 0 ug/ml
Conc. 5 ug/ml
Conc. 50 ug/ml

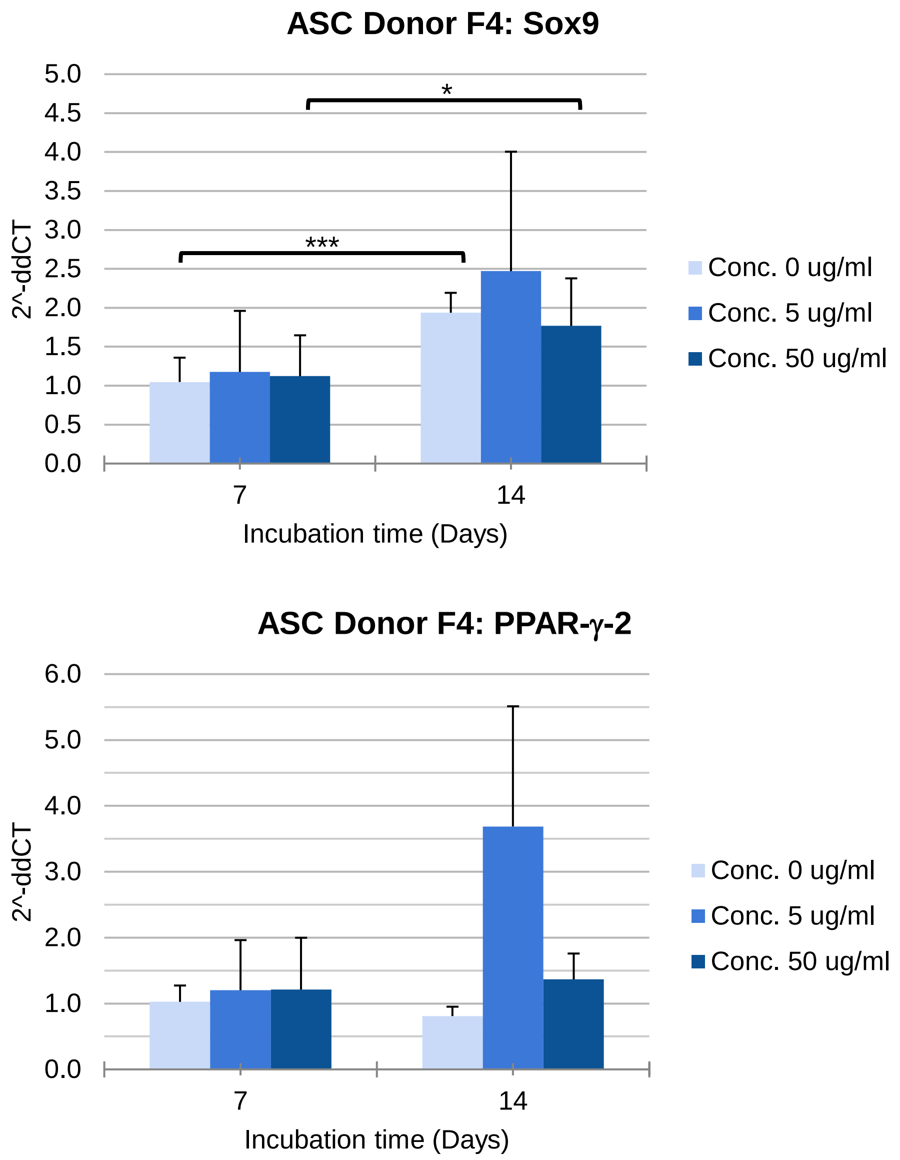

Figure S1. Average manifold induction of different genes for donor 1 (internal abbreviation F4). Experiments were carried out for three conditions and two time points, with 0, 5 or 50 ug/mL aCaP nanoparticles, denoted as Conc. = concentration and incubation for 7 or 14 days in culture, respectively.

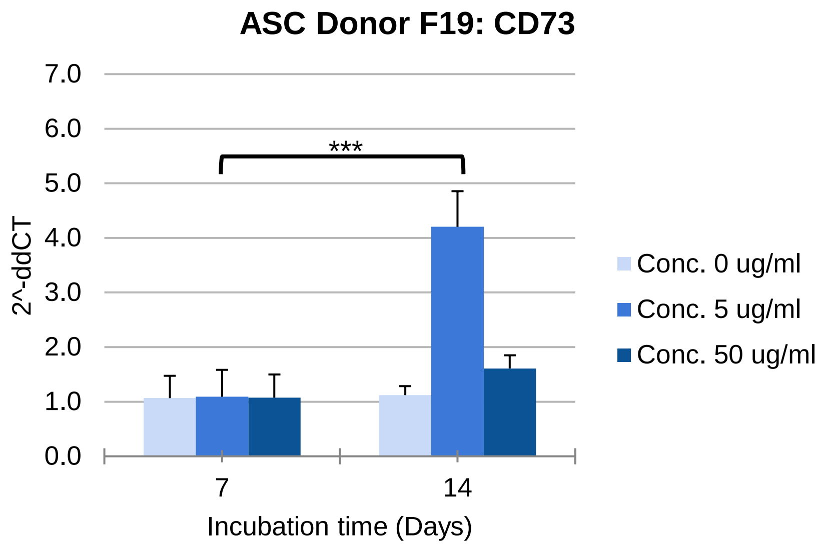
ASC Donor F19: CD73
7.0
6.0
5.0
4.0
3.0
2.0
1.0
0.0
2^-ddCT
***
7
14
Incubation time (Days)
Conc. 0 ug/ml
Conc. 5 ug/ml
Conc. 50 ug/ml

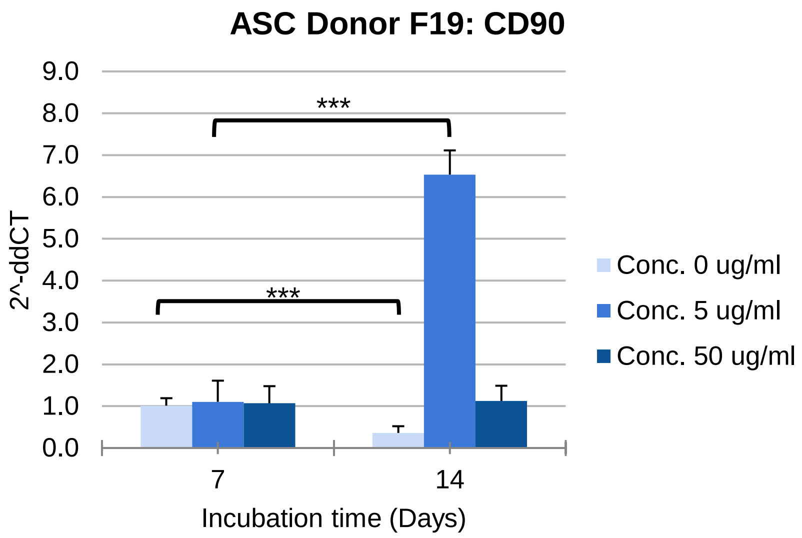
ASC Donor F19: CD90
9.0
8.0
7.0
6.0
5.0
4.0
3.0
2.0
1.0
0.0
2^-ddCT
***
***
7
14
Incubation time (Days)
Conc. 0 ug/ml
Conc. 5 ug/ml
Conc. 50 ug/ml

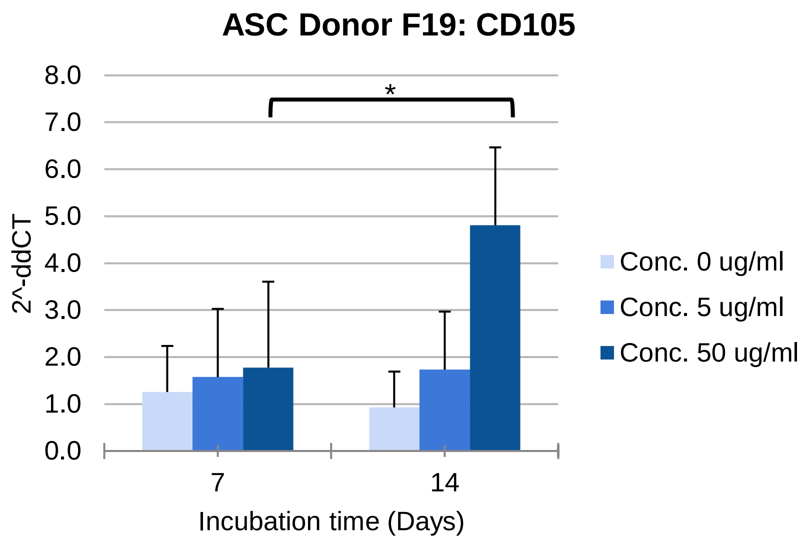
ASC Donor F19: CD105
8.0
7.0
6.0
5.0
4.0
3.0
2.0
1.0
0.0
2^-ddCT
*
7
14
Incubation time (Days)
Conc. 0 ug/ml
Conc. 5 ug/ml
Conc. 50 ug/ml

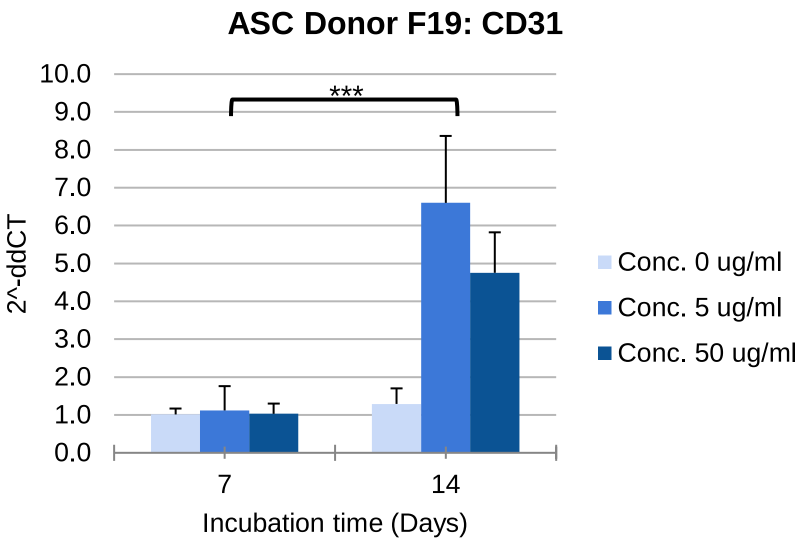
ASC Donor F19: CD31
***
10.0
9.0
8.0
7.0
6.0
5.0
4.0
3.0
2.0
1.0
0.0
2^-ddCT
7
14
Incubation time (Days)
Conc. 0 ug/ml
Conc. 5 ug/ml
Conc. 50 ug/ml

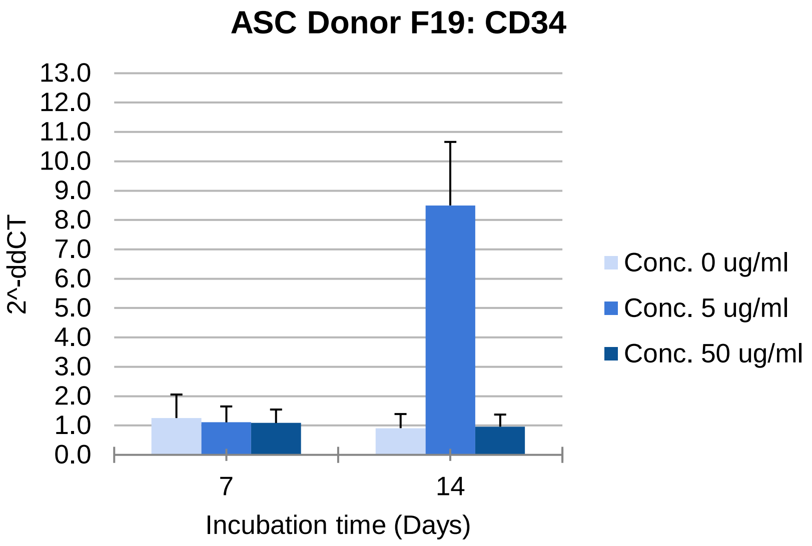
ASC Donor F19: CD34
13.0
12.0
11.0
10.0
9.0
8.0
7.0
6.0
5.0
4.0
3.0
2.0
1.0
0.0
2^-ddCT
7
14
Incubation time (Days)
Conc. 0 ug/ml
Conc. 5 ug/ml
Conc. 50 ug/ml

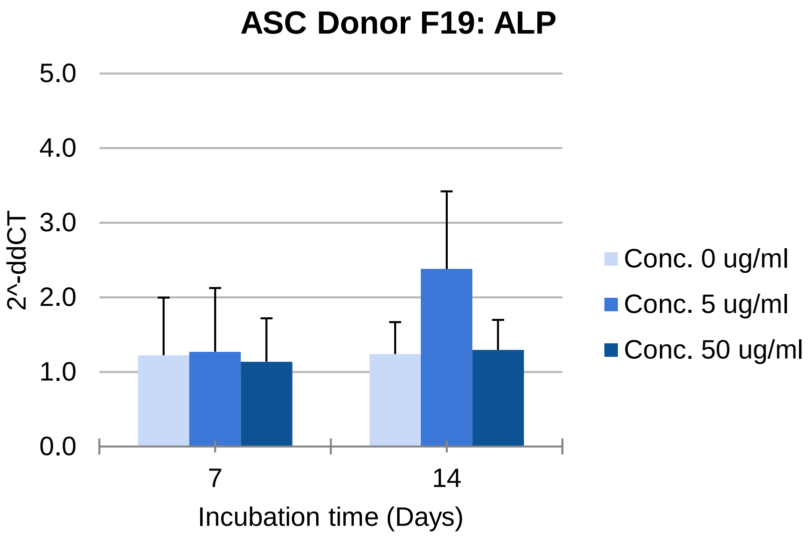
ASC Donor F19: ALP
5.0
4.0
3.0
2.0
1.0
0.0
2^-ddCT
7
14
Incubation time (Days)
Conc. 0 ug/ml
Conc. 5 ug/ml
Conc. 50 ug/ml

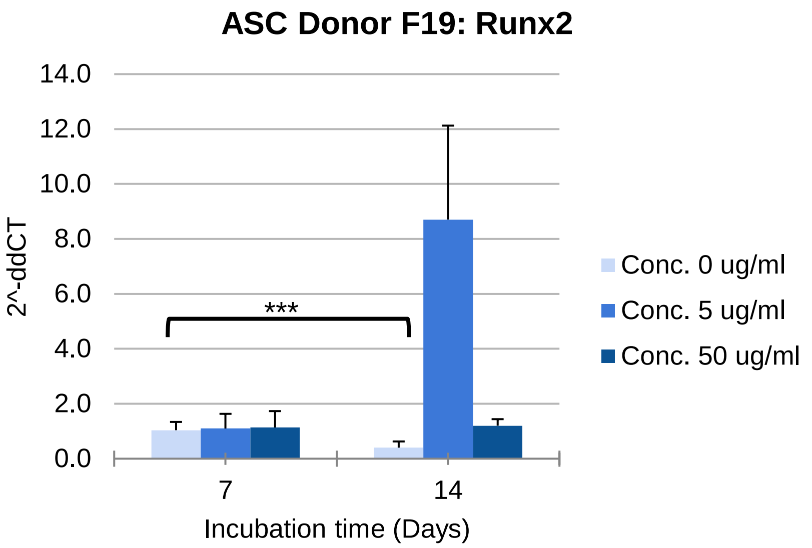
ASC Donor F19: Runx2
14.0
12.0
10.0
8.0
6.0
4.0
2.0
0.0
2^-ddCT
***
7
14
Incubation time (Days)
Conc. 0 ug/ml
Conc. 5 ug/ml
Conc. 50 ug/ml

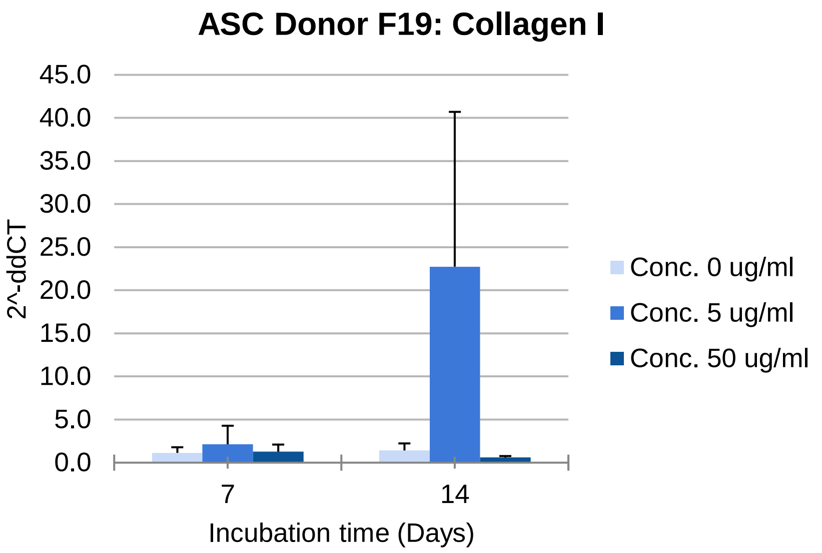
ASC Donor F19: Collagen I
45.0
40.0
35.0
30.0
25.0
20.0
15.0
10.0
5.0
0.0
2^-ddCT
7
14
Incubation time (Days)
Conc. 0 ug/ml
Conc. 5 ug/ml
Conc. 50 ug/ml

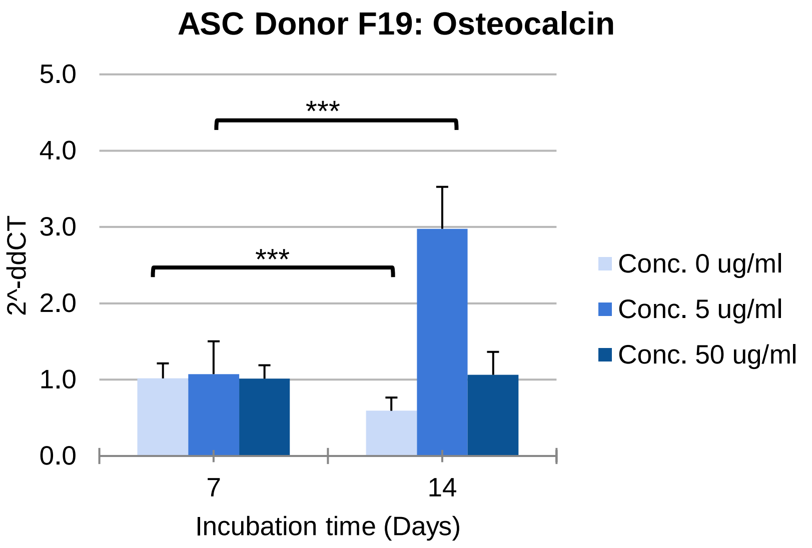
ASC Donor F19: Osteocalcin
5.0
4.0
3.0
2.0
1.0
0.0
2^-ddCT
***
***
7
14
Incubation time (Days)
Conc. 0 ug/ml
Conc. 5 ug/ml
Conc. 50 ug/ml

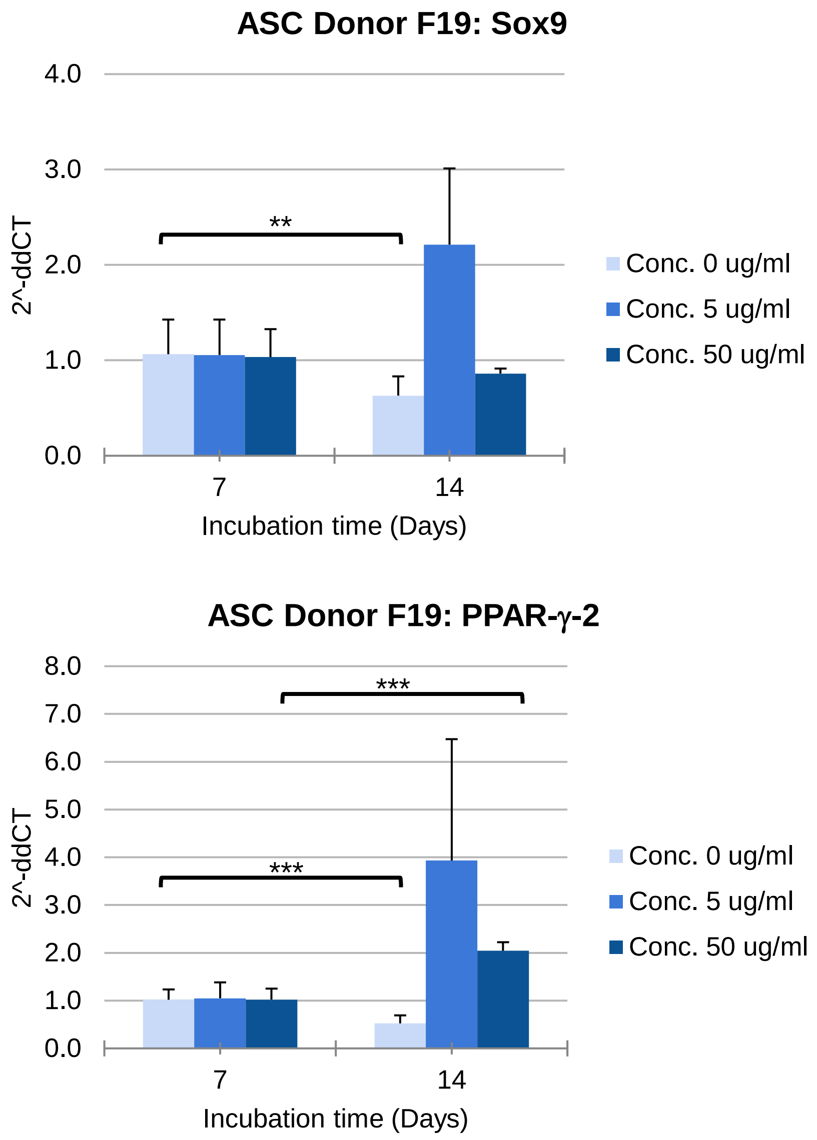

Figure S2. Average manifold induction of different genes for donor 2 (internal abbreviation F19). Experiments were carried out for three conditions and two time points, with 0, 5 or 50 ug/mL aCaP nanoparticles, denoted as Conc. = concentration and incubation for 7 or 14 days in culture, respectively.

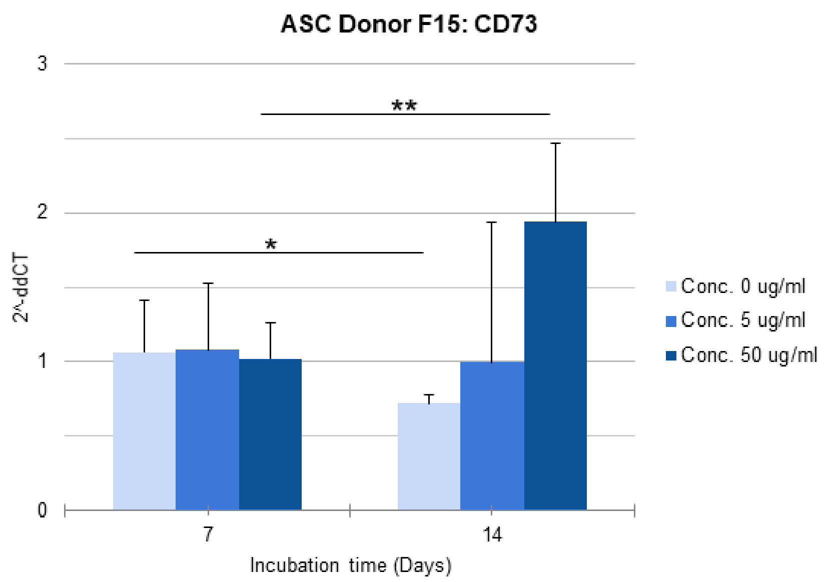
ASC Donor F15: CD73
3
2
1
0
2^-ddCT
**
*
7
14
Incubation time (Days)
Conc. 0 ug/ml
Conc. 5 ug/ml
Conc. 50 ug/ml

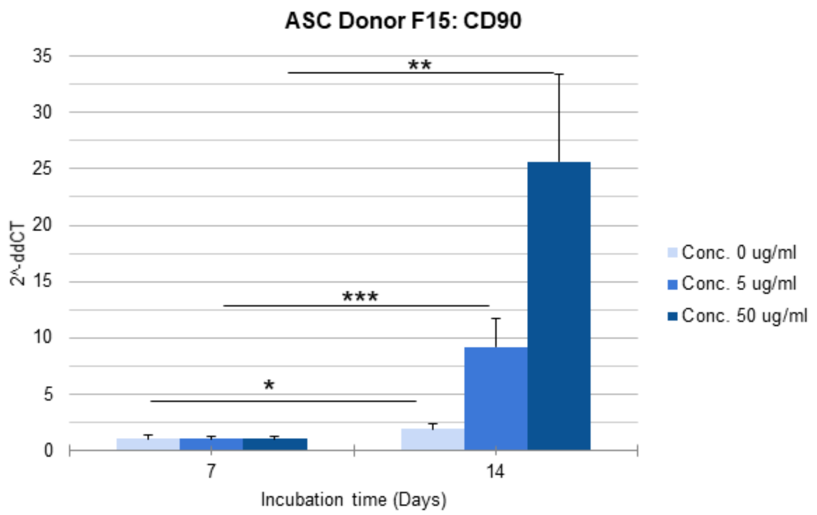
ASC Donor F15: CD90
35
30
25
20
15
10
5
0
2^-ddCT
**
***
*
7
14
Incubation time (Days)
Conc. 0 ug/ml
Conc. 5 ug/ml
Conc. 50 ug/ml

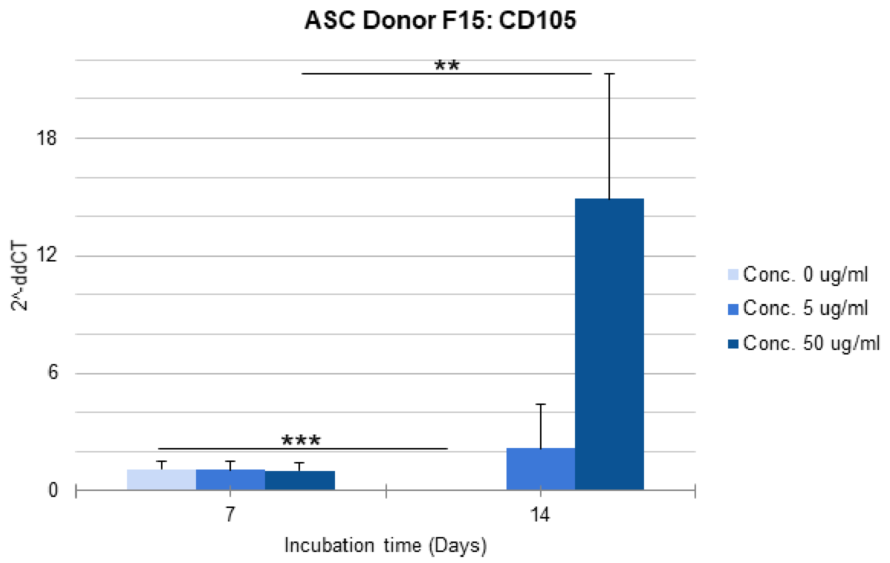
ASC Donor F15: CD105
18
12
6
0
2^-ddCT
**
***
7
14
Incubation time (Days)
Conc. 0 ug/ml
Conc. 5 ug/ml
Conc. 50 ug/ml

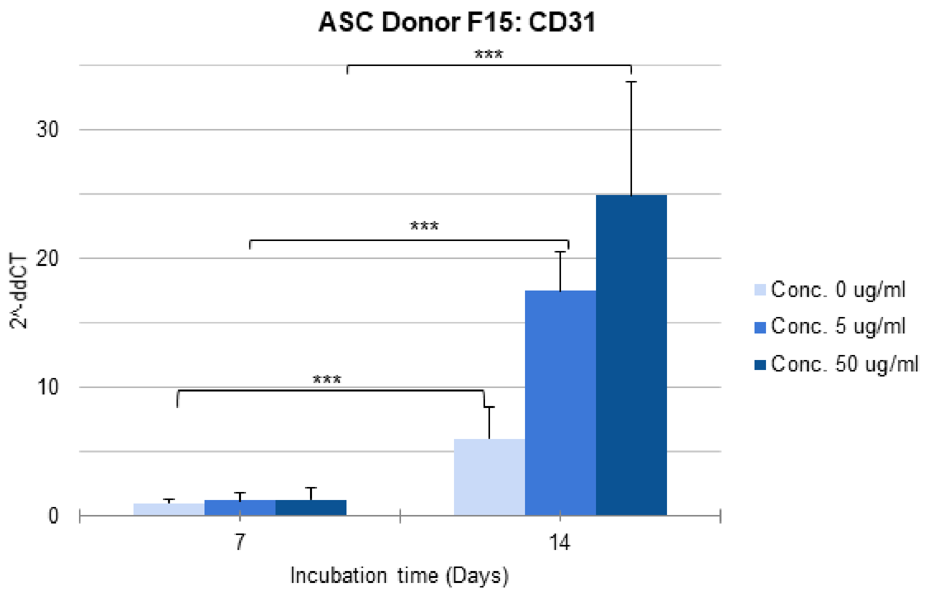

ASC Donor F15: CD31
***
***
***
30
20
10
0
2^-ddCT
7
14
Incubation time (Days)
Conc. 0 ug/ml
Conc. 5 ug/ml
Conc. 50 ug/ml

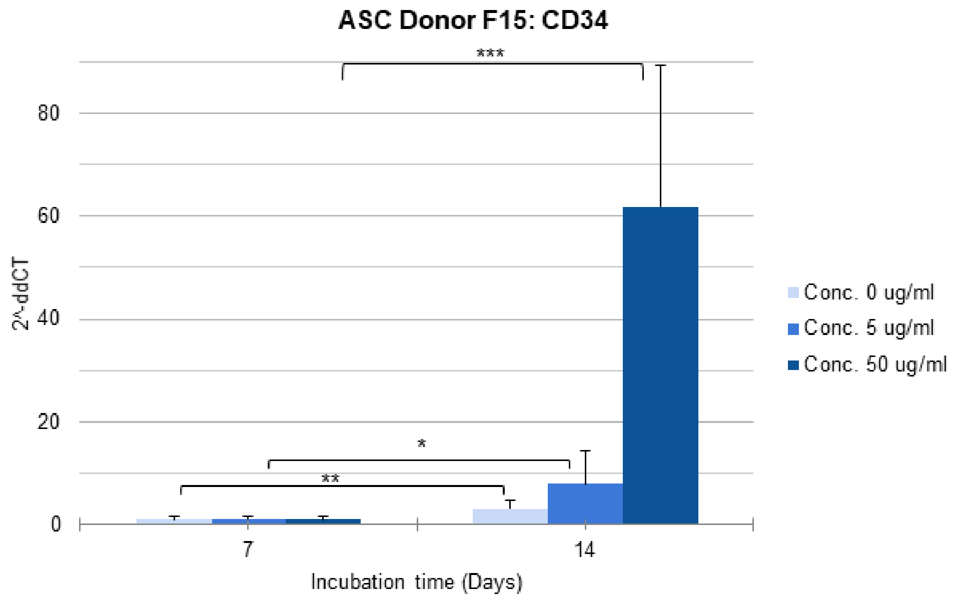

ASC Donor F15: CD34
***
*
**
80
60
40
20
0
2^-ddCT
7
14
Incubation time (Days)
Conc. 0 ug/ml
Conc. 5 ug/ml
Conc. 50 ug/ml

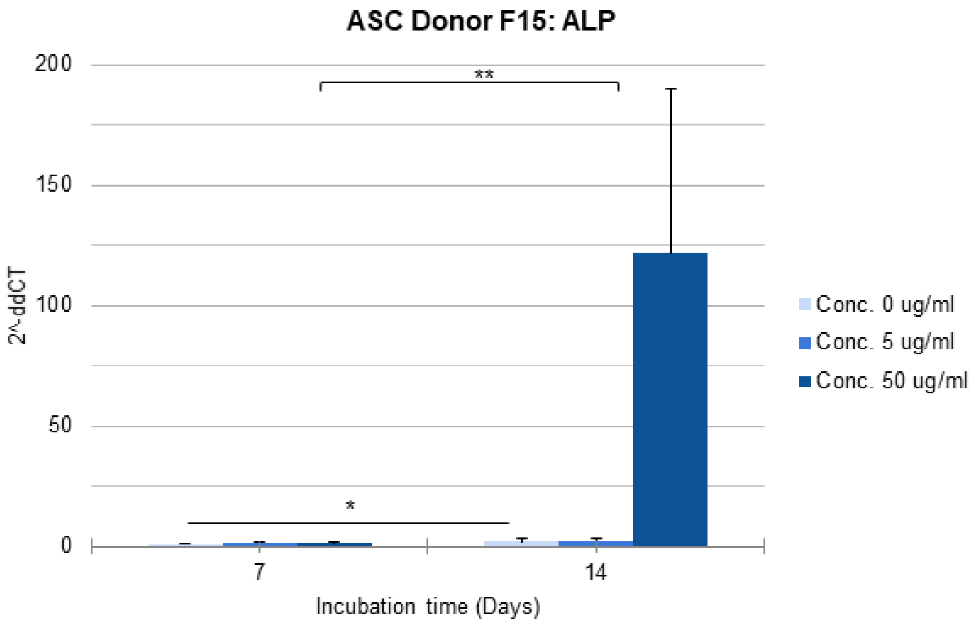
ASC Donor F15: ALP
200
150
100
50
0
2^-ddCT
**
*
7
14
Incubation time (Days)
Conc. 0 ug/ml
Conc. 5 ug/ml
Conc. 50 ug/ml

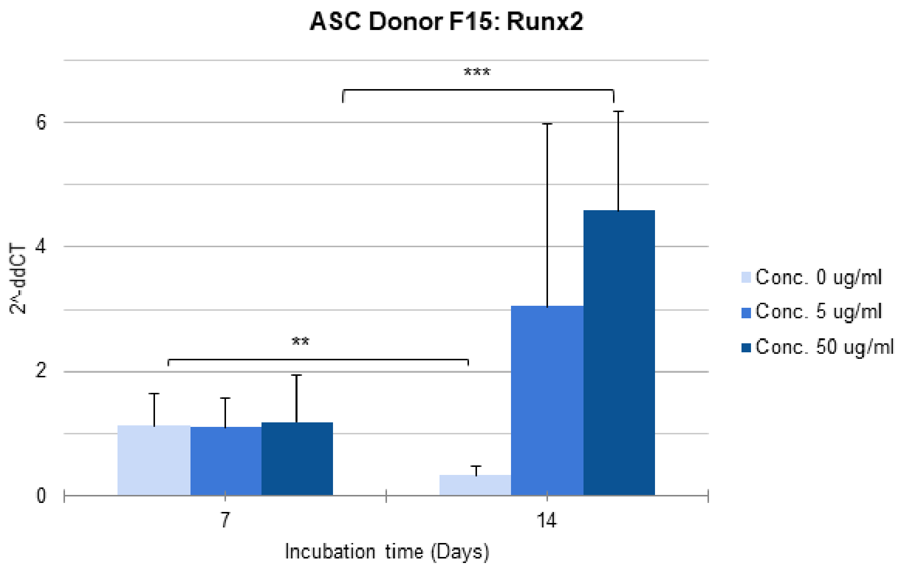
ASC Donor F15: Runx2
6
4
2
0
2^-ddCT
***
**
7
14
Incubation time (Days)
Conc. 0 ug/ml
Conc. 5 ug/ml
Conc. 50 ug/ml

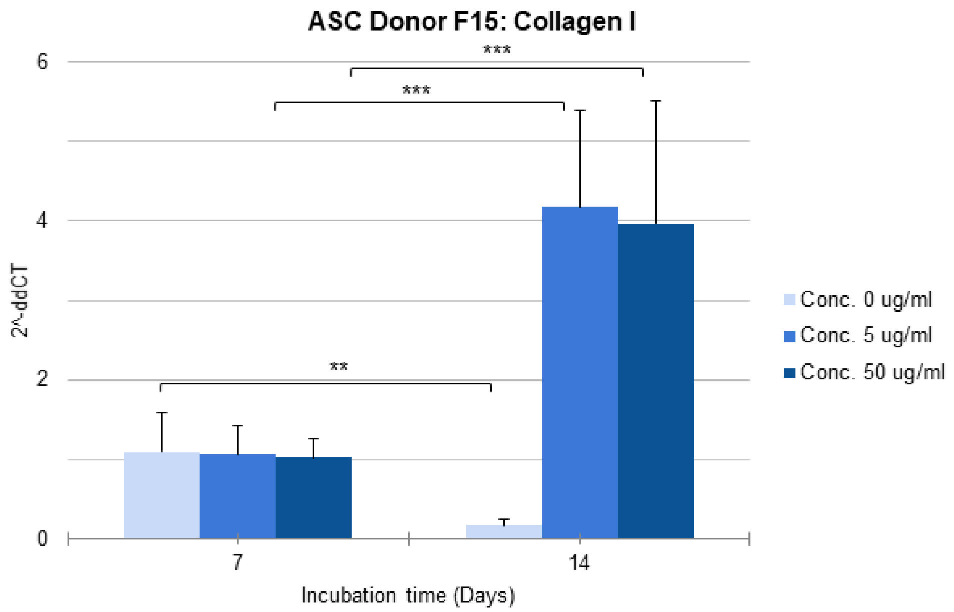
ASC Donor F15: Collagen I
6
4
2
0
2^-ddCT
***
***
**
7
14
Incubation time (Days)
Conc. 0 ug/ml
Conc. 5 ug/ml
Conc. 50 ug/ml

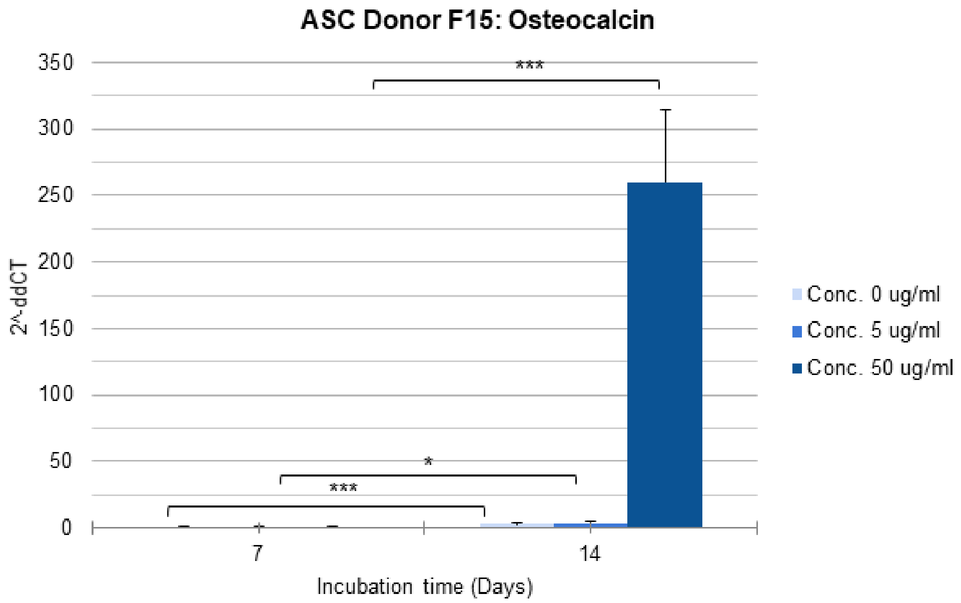
ASC Donor F15: Osteocalcin
350
300
250
200
150
100
50
0
2^-ddCT
***
*
***
7
14
Incubation time (Days)
Conc. 0 ug/ml
Conc. 5 ug/ml
Conc. 50 ug/ml

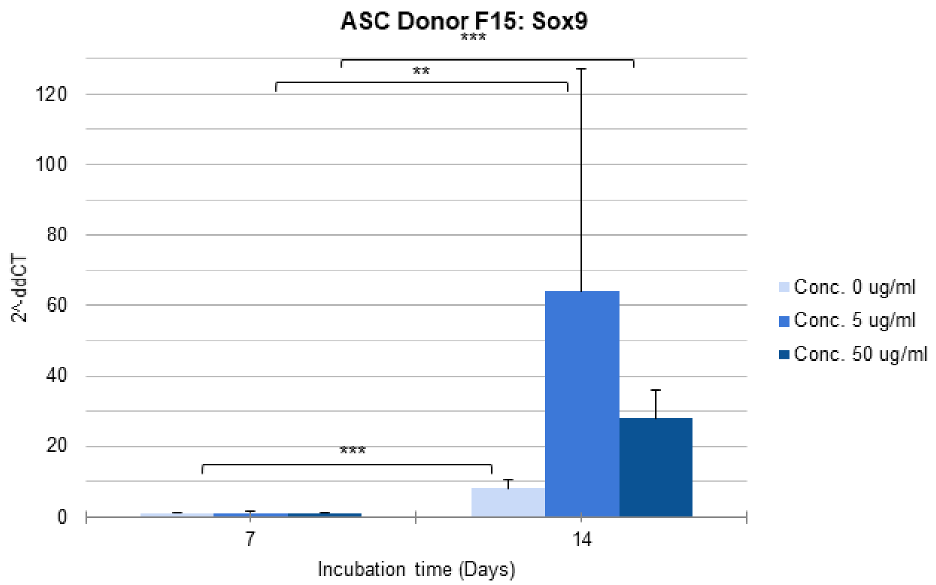
ASC Donor F15: Sox9
***
**
120
100
80
60
40
20
0
2^-ddCT
***
7
14
Incubation time (Days)
Conc. 0 ug/ml
Conc. 5 ug/ml
Conc. 50 ug/ml

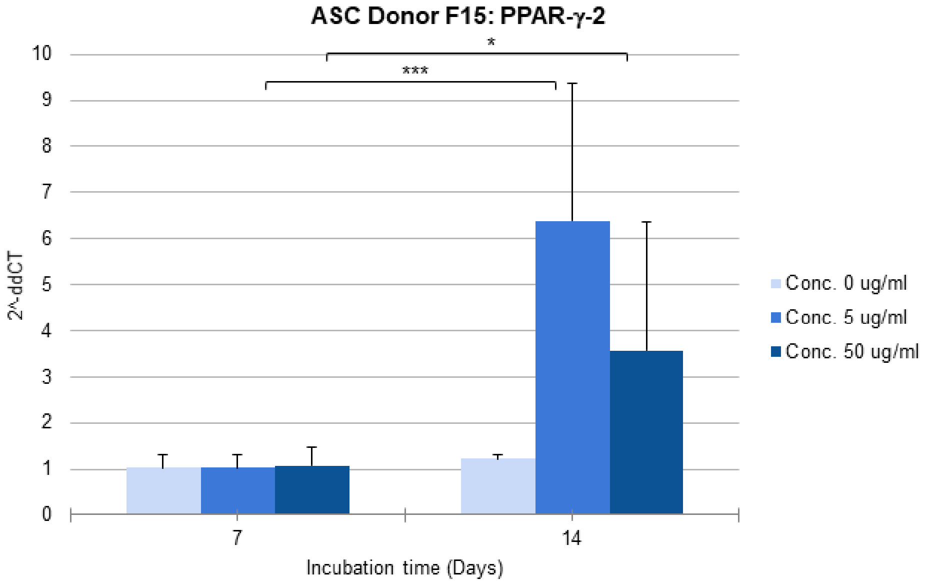

Figure S3. Average manifold induction of different genes for donor 3 (internal abbreviation F15). Experiments were carried out for three conditions and two time points, with 0, 5 or 50 ug/mL aCaP nanoparticles, denoted as Conc. = concentration and incubation for 7 or 14 days in culture, respectively.

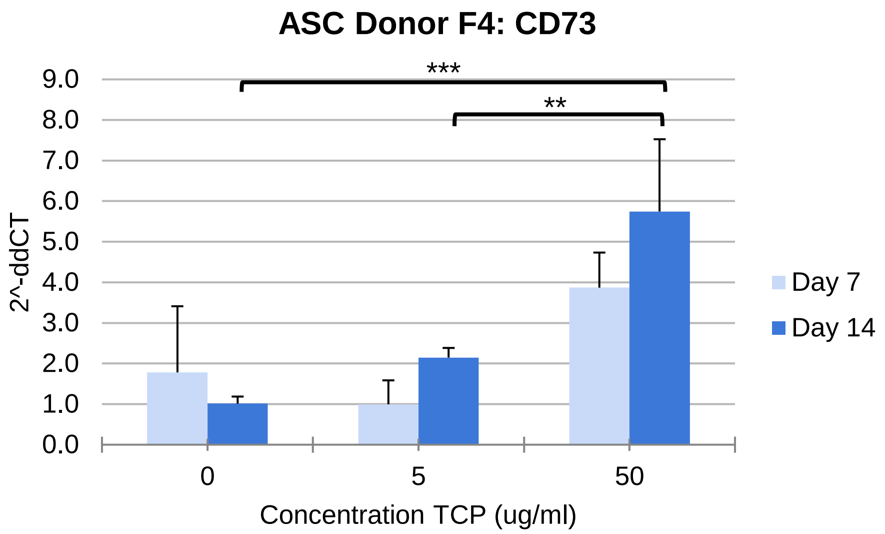
ASC Donor F4: CD73
***
**
9.0
8.0
7.0
6.0
5.0
4.0
3.0
2.0
1.0
0.0
2^-ddCT
0
5
50
Concentration TCP (ug/ml)
Day 7
Day 14

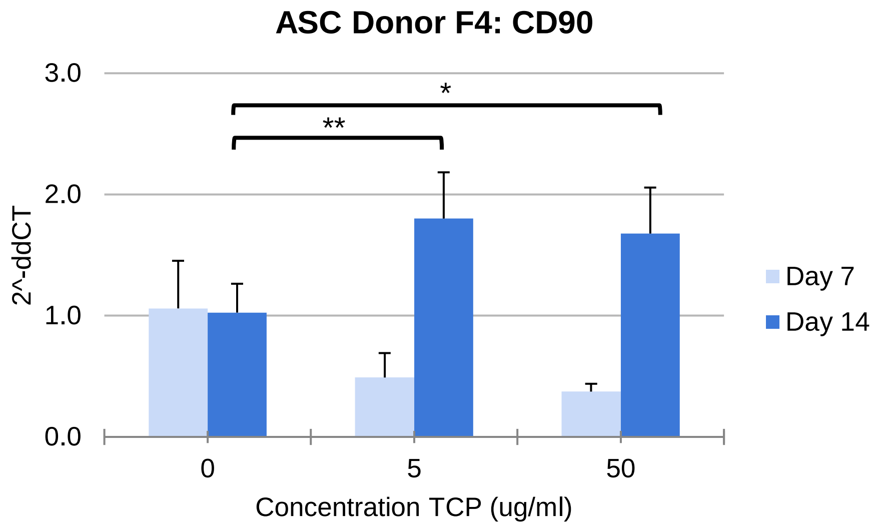
ASC Donor F4: CD90
*
**
3.0
2.0
1.0
0.0
2^-ddCT
0
5
50
Concentration TCP (ug/ml)
Day 7
Day 14

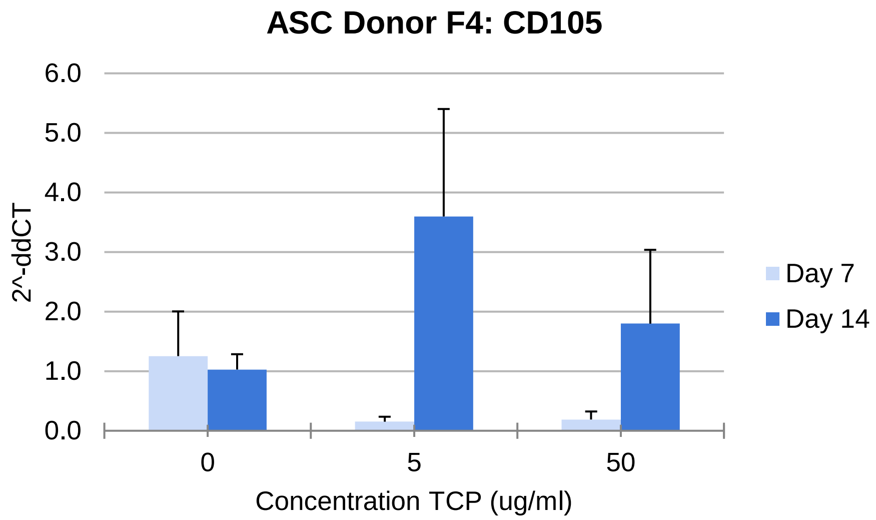
ASC Donor F4: CD105
6.0
5.0
4.0
3.0
2.0
1.0
0.0
2^-ddCT
0
5
50
Concentration TCP (ug/ml)
Day 7
Day 14

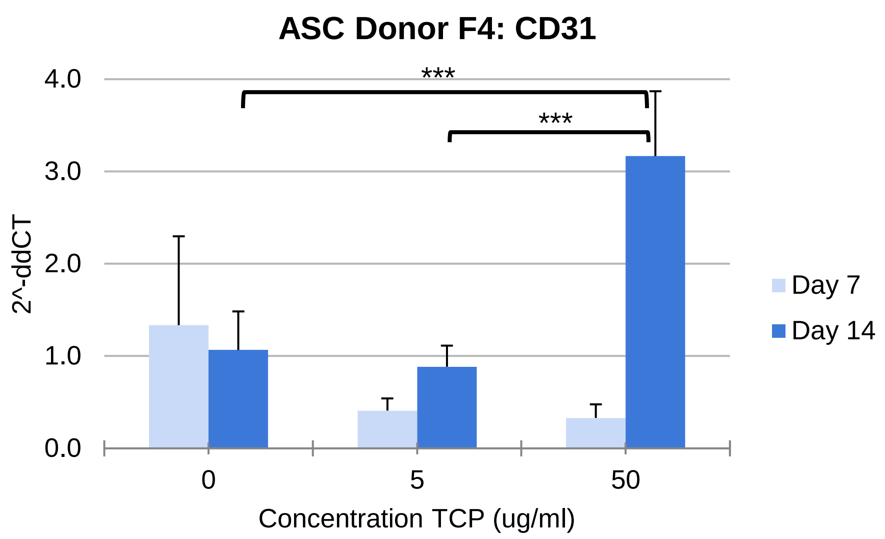
ASC Donor F4: CD31
4.0
3.0
2.0
1.0
0.0
2^-ddCT
***
***
0
5
50
Concentration TCP (ug/ml)
Day 7
Day 14

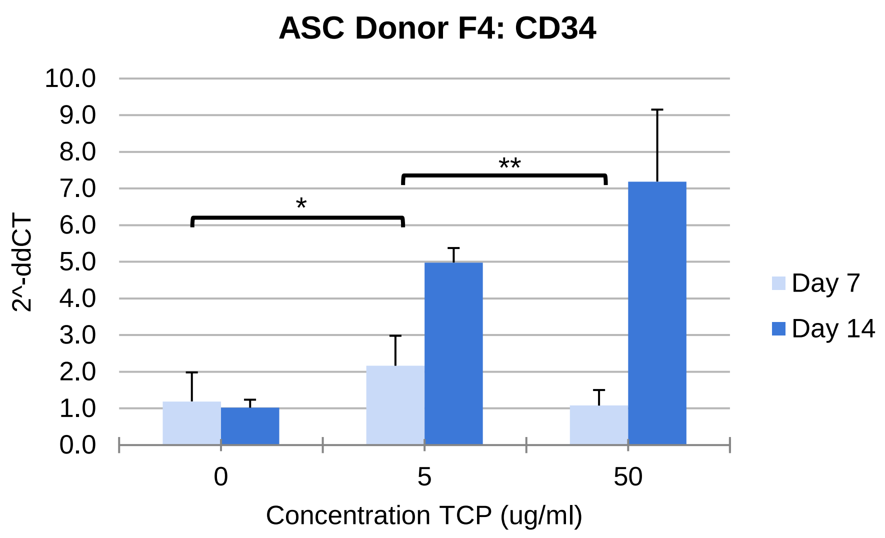
ASC Donor F4: CD34
10.0
9.0
8.0
7.0
6.0
5.0
4.0
3.0
2.0
1.0
0.0
2^-ddCT
*
**
0
5
50
Concentration TCP (ug/ml)
Day 7
Day 14

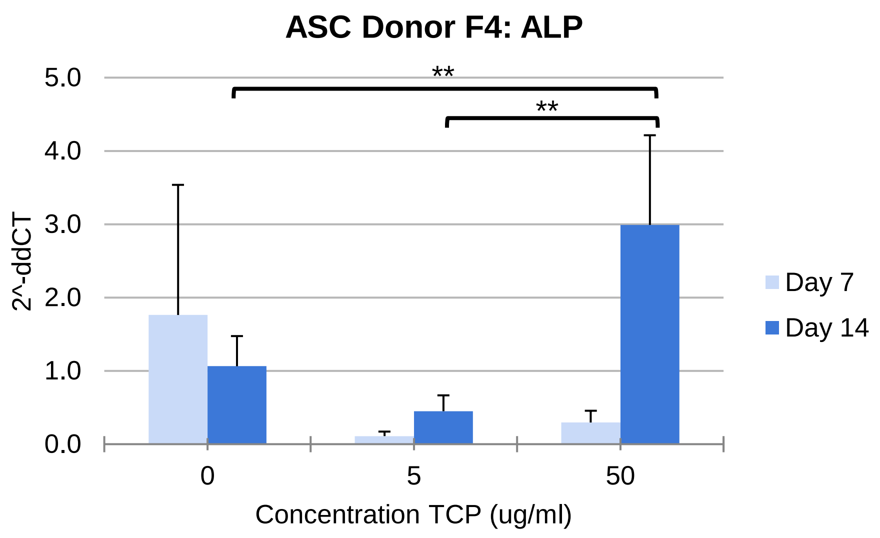
ASC Donor F4: ALP
5.0
4.0
3.0
2.0
1.0
0.0
2^-ddCT
**
**
0
5
50
Concentration TCP (ug/ml)
Day 7
Day 14

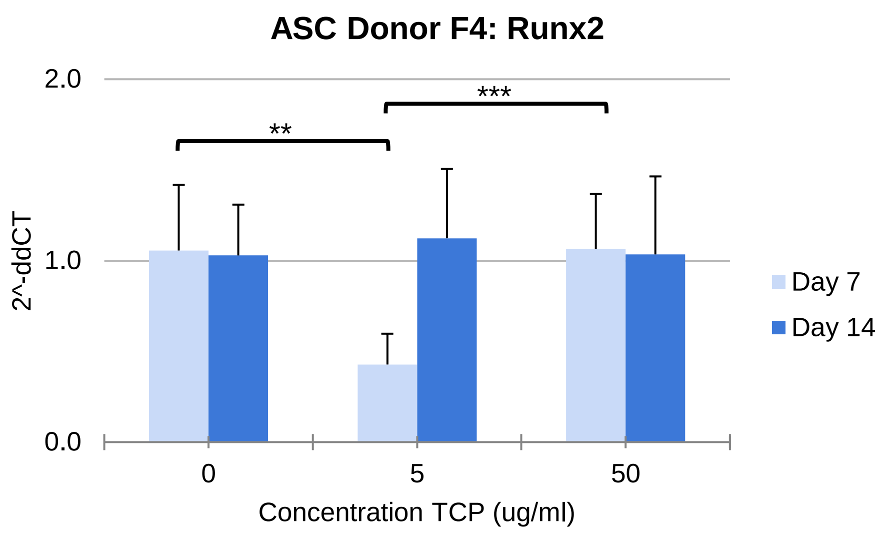
ASC Donor F4: Runx2
2.0
1.0
0.0
2^-ddCT
**
***
0
5
50
Concentration TCP (ug/ml)
Day 7
Day 14

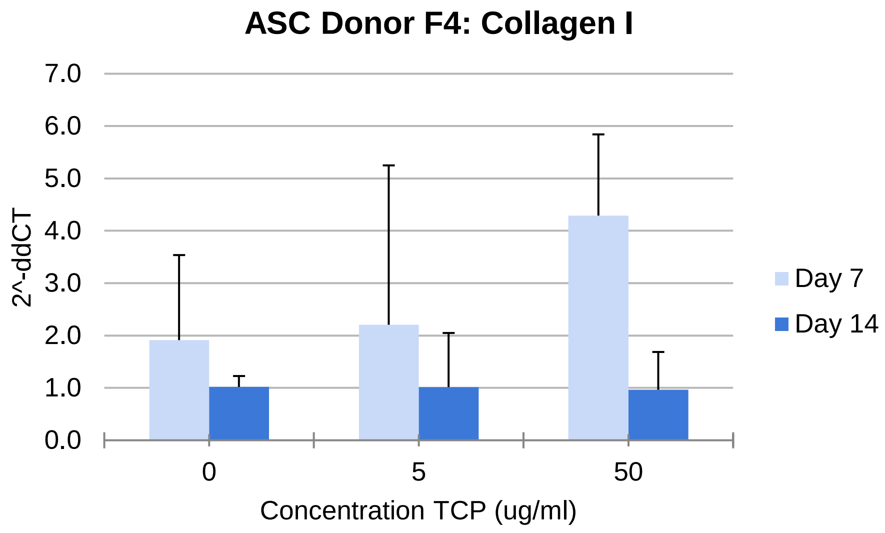
ASC Donor F4: Collagen I
7.0
6.0
5.0
4.0
3.0
2.0
1.0
0.0
2^-ddCT
0
5
50
Concentration TCP (ug/ml)
Day 7
Day 14

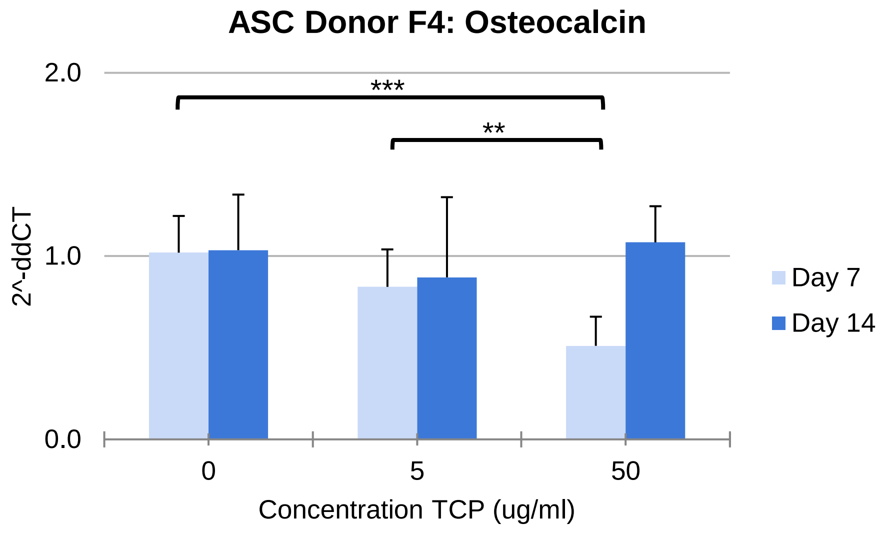
ASC Donor F4: Osteocalcin
2.0
1.0
0.0
2^-ddCT
***
**
0
5
50
Concentration TCP (ug/ml)
Day 7
Day 14

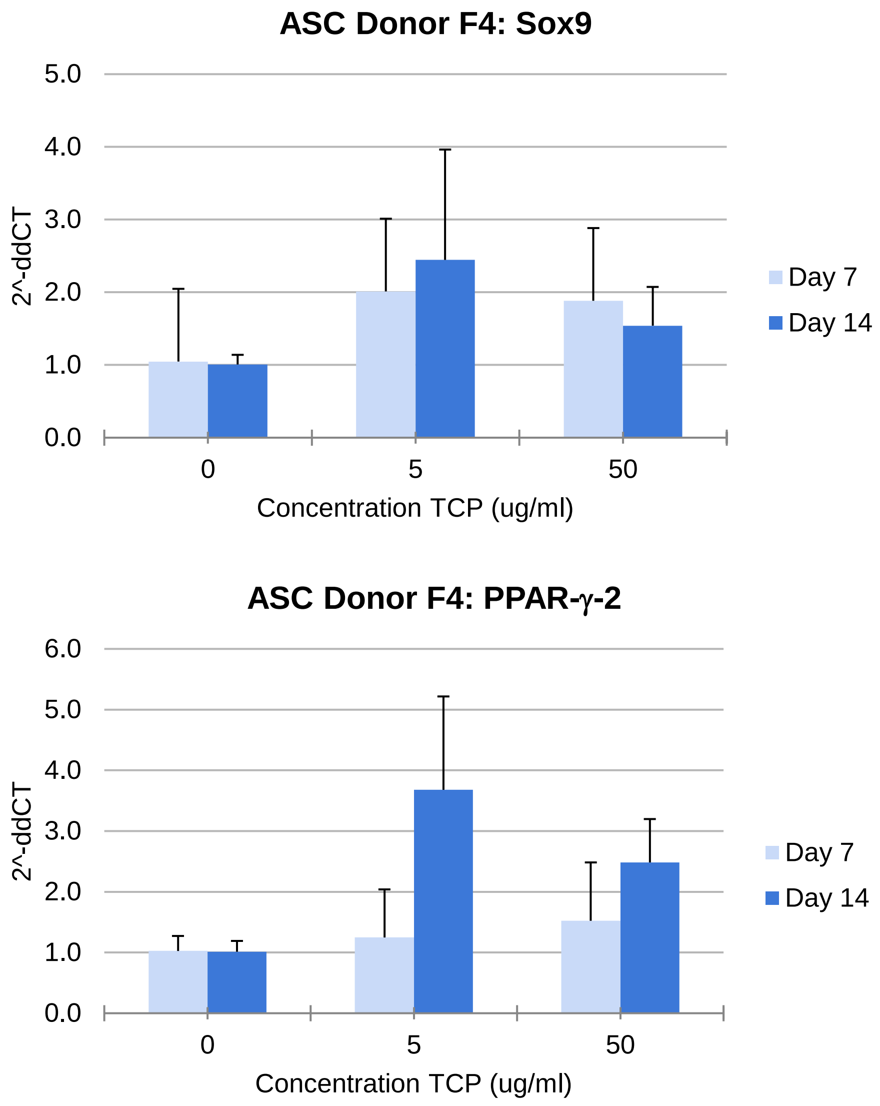

Figure S4. Average manifold induction of different genes for donor 1 (internal abbreviation F4). Experiments were carried out for three conditions and two time points, with 0, 5 or 50 ug/mL aCaP nanoparticles and at 7 or 14 days in culture, respectively. Key: TCP = amorphous calcium phosphate nanoparticles.

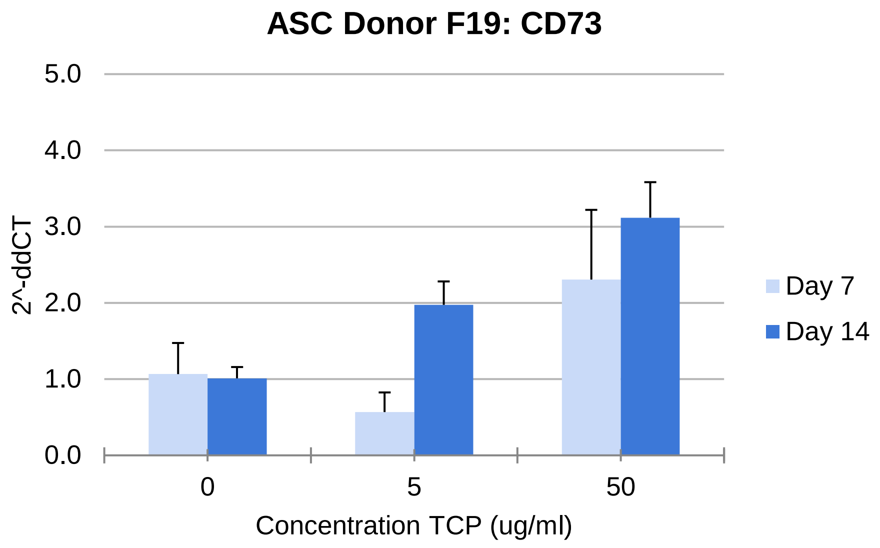
ASC Donor F19: CD73
5.0
4.0
3.0
2.0
1.0
0.0
2^-ddCT
0
5
50
Concentration TCP (ug/ml)
Day 7
Day 14

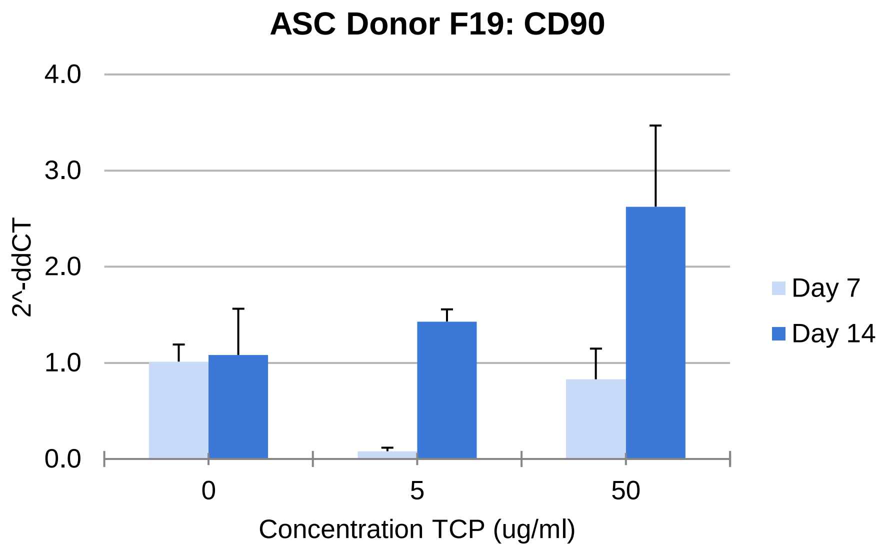
ASC Donor F19: CD90
4.0
3.0
2.0
1.0
0.0
2^-ddCT
0
5
50
Concentration TCP (ug/ml)
Day 7
Day 14

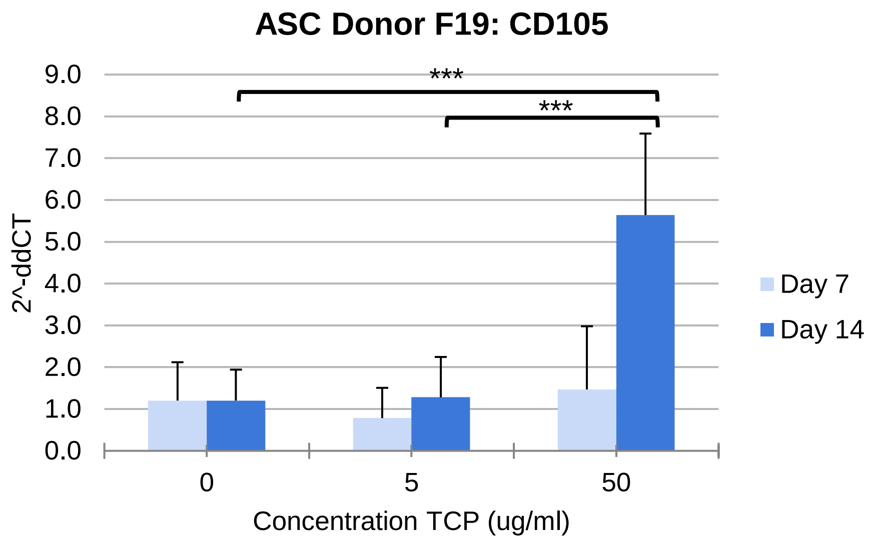
ASC Donor F19: CD105
***
***
9.0
8.0
7.0
6.0
5.0
4.0
3.0
2.0
1.0
0.0
2^-ddCT
0
5
50
Concentration TCP (ug/ml)
Day 7
Day 14

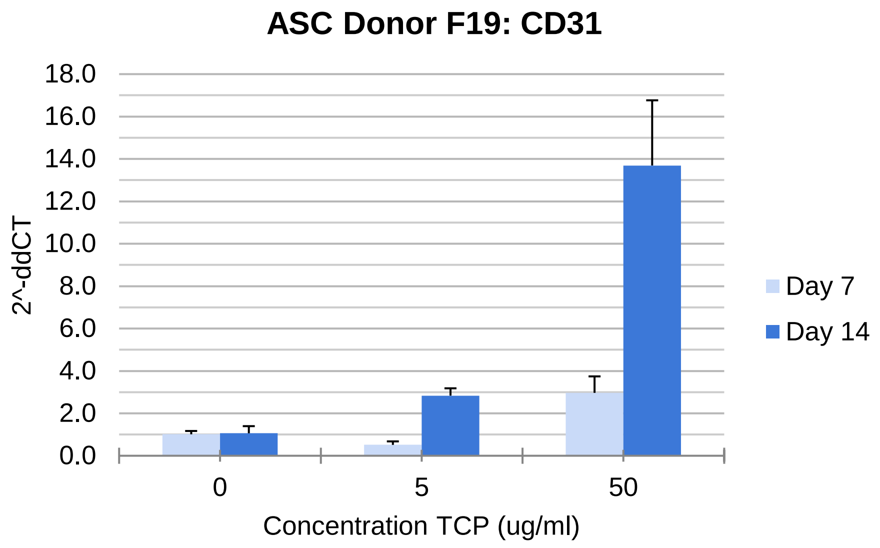
ASC Donor F19: CD31
18.0
16.0
14.0
12.0
10.0
8.0
6.0
4.0
2.0
0.0
2^-ddCT
0
5
50
Concentration TCP (ug/ml)
Day 7
Day 14

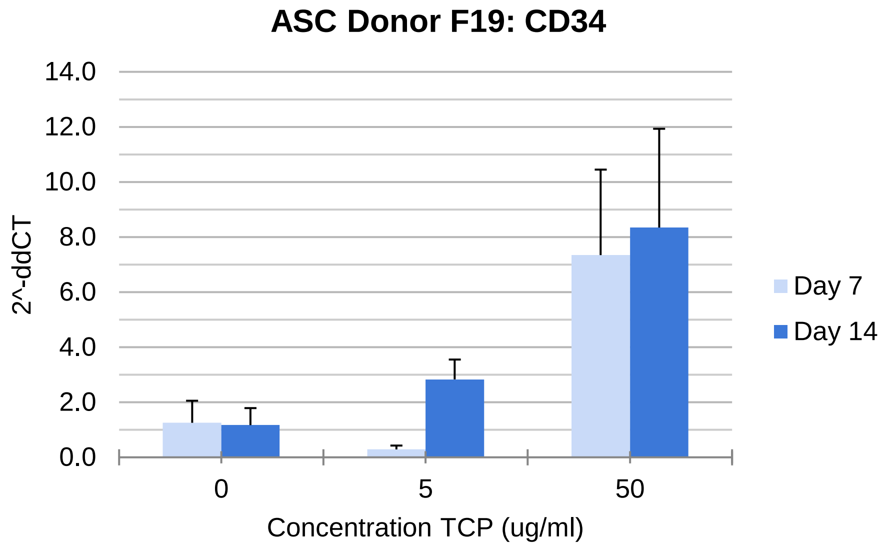
ASC Donor F19: CD34
14.0
12.0
10.0
8.0
6.0
4.0
2.0
0.0
2^-ddCT
0
5
50
Concentration TCP (ug/ml)
Day 7
Day 14

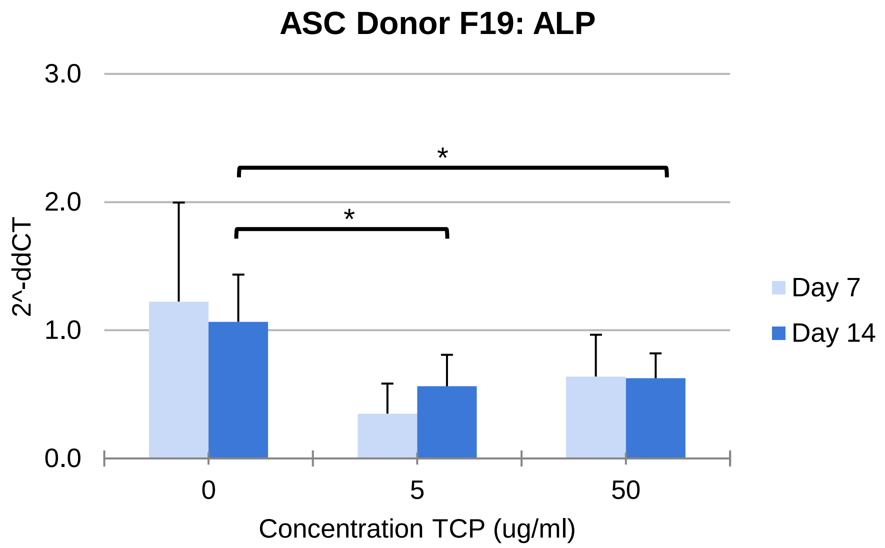
ASC Donor F19: ALP
3.0
2.0
1.0
0.0
2^-ddCT
*
*
0
5
50
Concentration TCP (ug/ml)
Day 7
Day 14

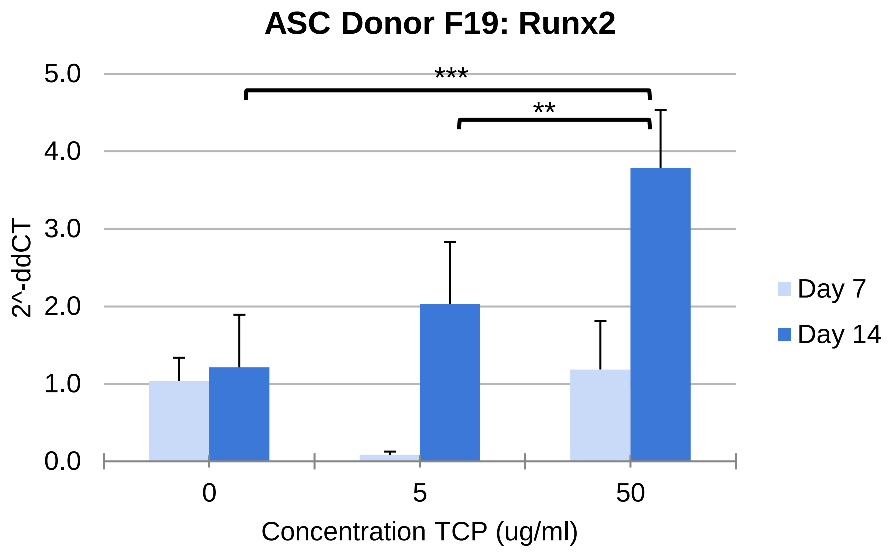
ASC Donor F19: Runx2
5.0
4.0
3.0
2.0
1.0
0.0
2^-ddCT
***
**
Day 7
Day 14
0
5
50
Concentration TCP (ug/ml)

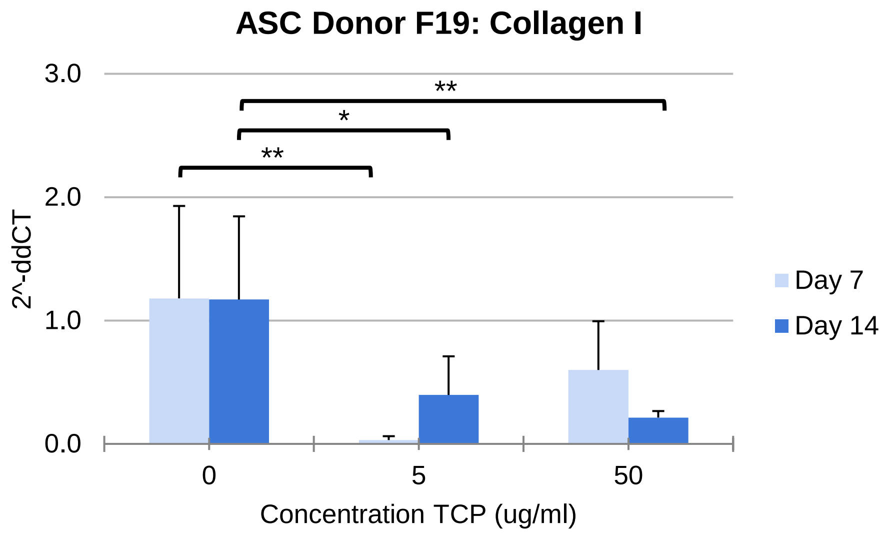
ASC Donor F19: Collagen I
3.0
2.0
1.0
0.0
2^-ddCT
**
*
**
Day 7
Day 14
0
5
50
Concentration TCP (ug/ml)

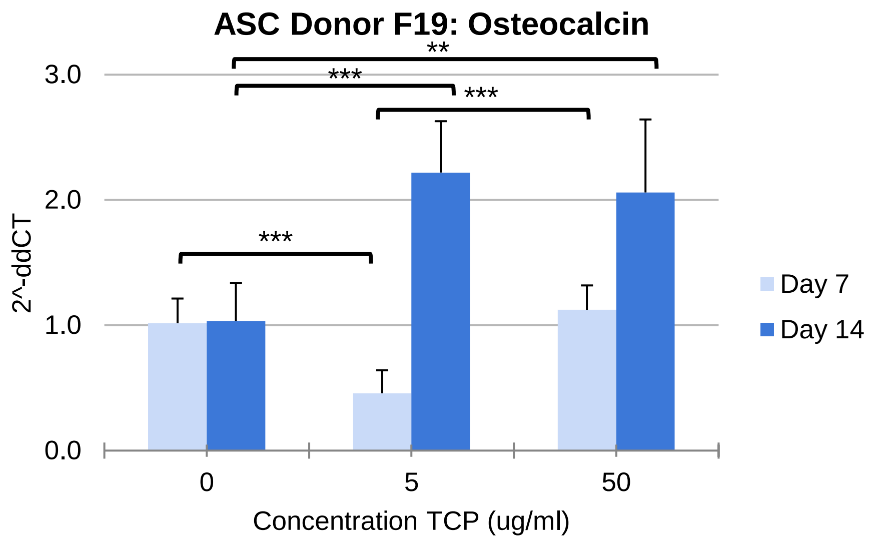
ASC Donor F19: Osteocalcin
3.0
2.0
1.0
0.0
2^-ddCT
**
***
***
***
Day 7
Day 14
0
5
50
Concentration TCP (ug/ml)

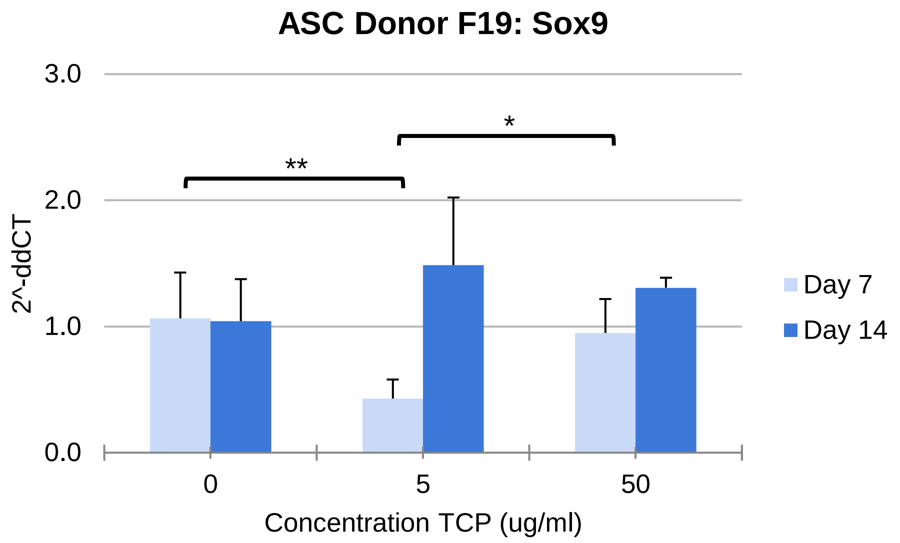

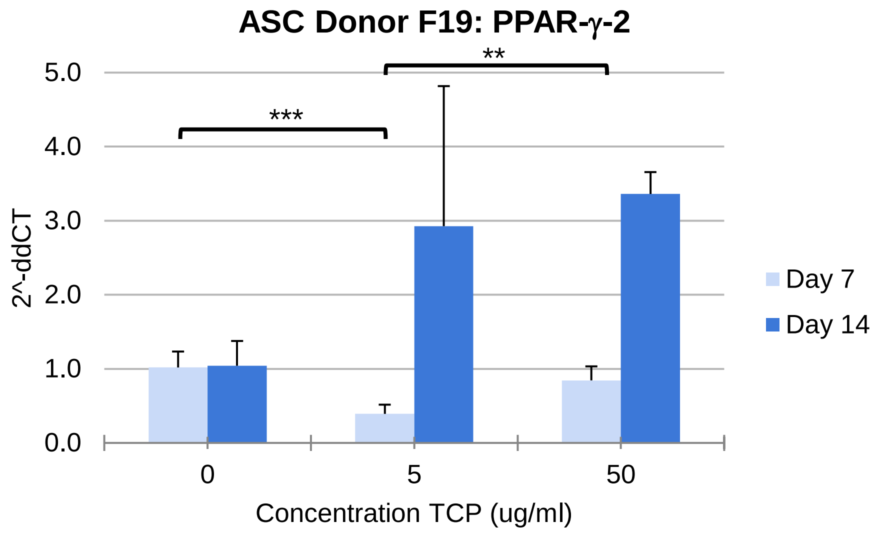

Figure S5. Average manifold induction of different genes for donor 2 (internal abbreviation F19). Experiments were carried out for three conditions and two time points, with 0, 5 or 50 ug/mL aCaP nanoparticles and at 7 or 14 days in culture, respectively. Key: TCP = amorphous calcium phosphate nanoparticles.

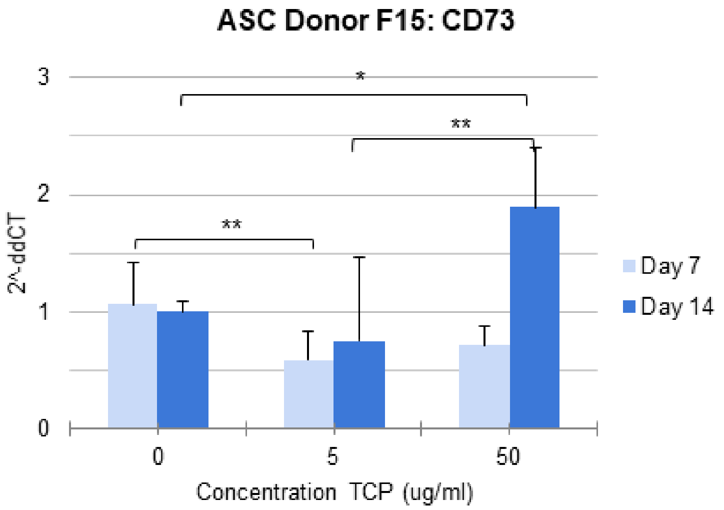
ASC Donor F15: CD73
3
2
1
0
2^-ddCT
*
**
**
0
5
50
Concentration TCP (ug/ml)
Day 7
Day 14

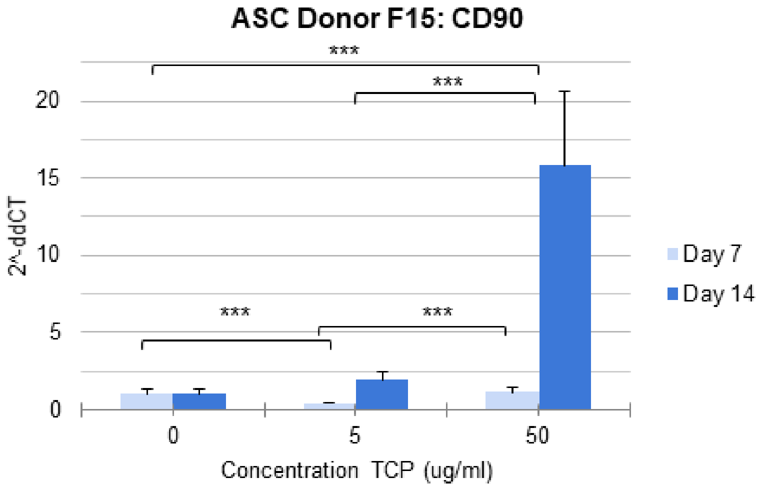
ASC Donor F15: CD90
20
15
10
5
0
2^-ddCT
***
***
***
***
0
5
50
Concentration TCP (ug/ml)
Day 7
Day 14

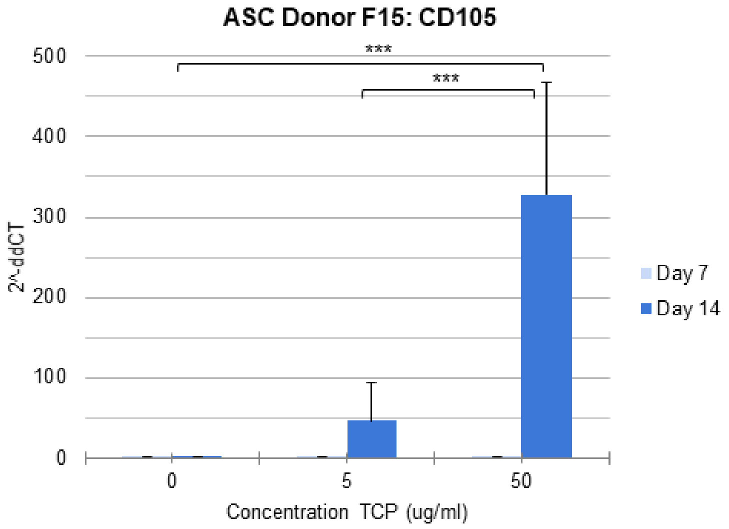
ASC Donor F15: CD105
500
400
300
200
100
0
2^-ddCT
***
***
0
5
50
Concentration TCP (ug/ml)
Day 7
Day 14

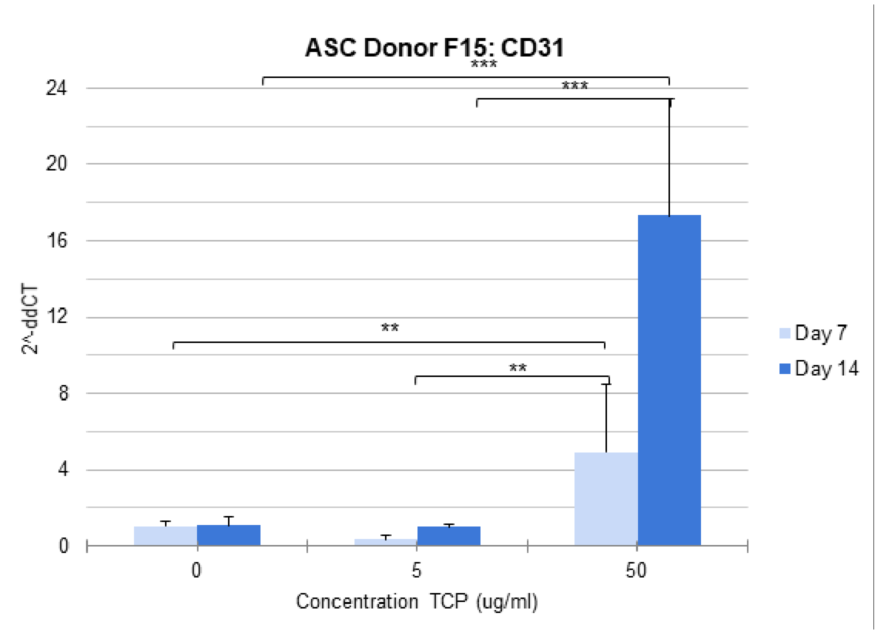
ASC Donor F15: CD31
***
***
**
**
2^-ddCT
24
20
16
12
8
4
0
0
5
50
Concentration TCP (ug/ml)
Day 7
Day 14

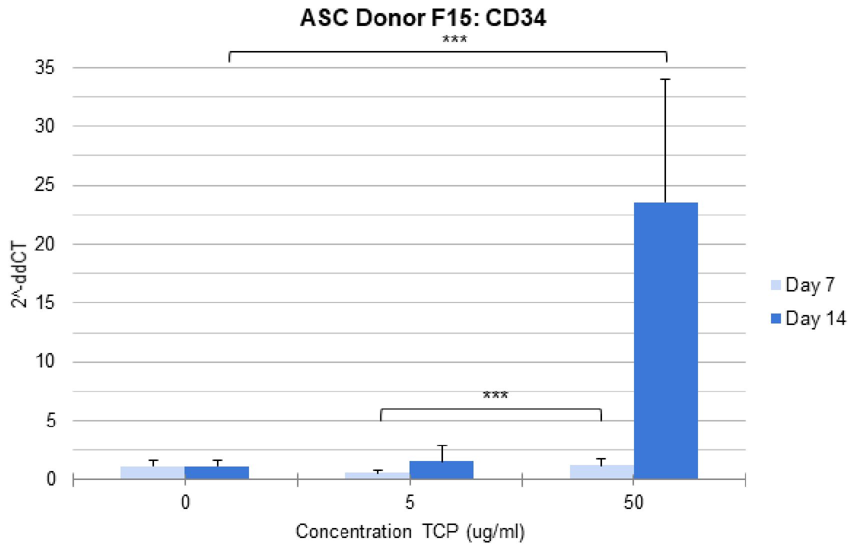
ASC Donor F15: CD34
***
***
2^-ddCT
35
30
25
20
15
10
5
0
0
5
50
Concentration TCP (ug/ml)
Day 7
Day 14

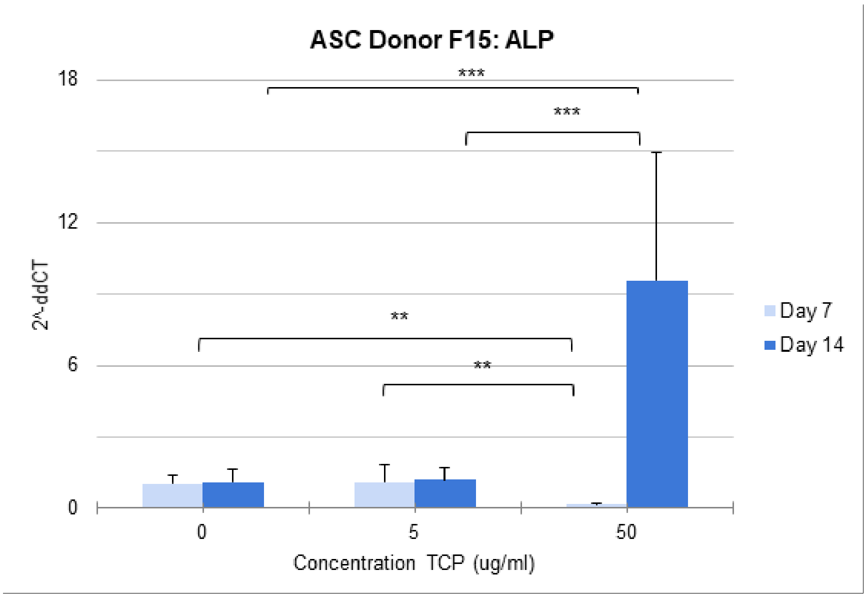
ASC Donor F15: ALP
***
***
**
**
2^-ddCT
18
12
6
0
0
5
50
Concentration TCP (ug/ml)
Day 7
Day 14

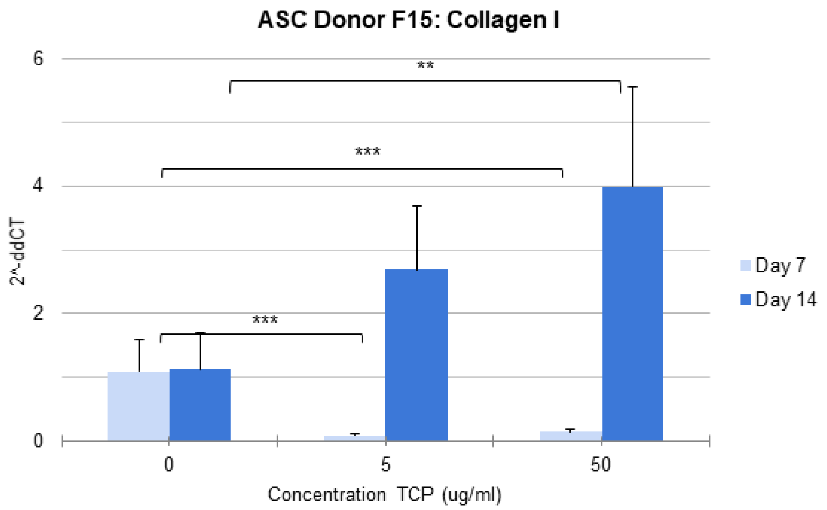
ASC Donor F15: Collagen I
2^-ddCT
6
4
2
0
**
***
***
Day 7
Day 14
0
5
50
Concentration TCP (ug/ml)

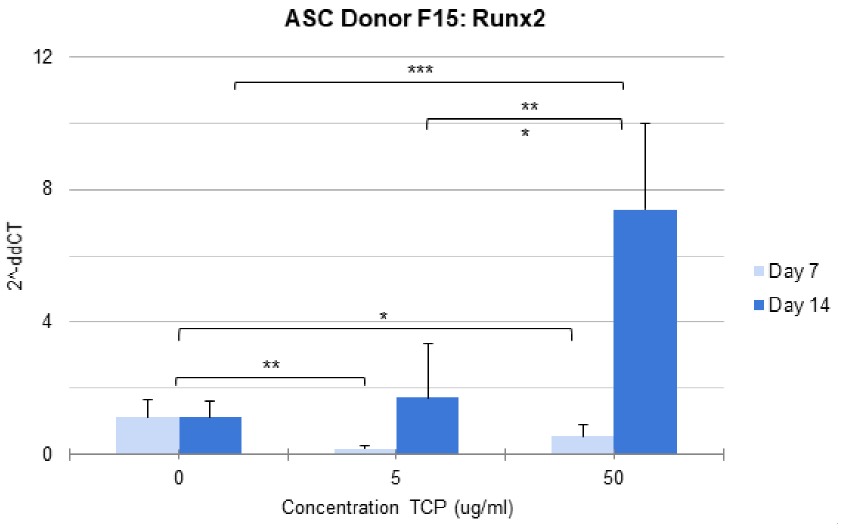
ASC Donor F15: Runx2
2^-ddCT
12
8
4
0
***
**
*
*
**
Day 7
Day 14
0
5
50
Concentration TCP (ug/ml)

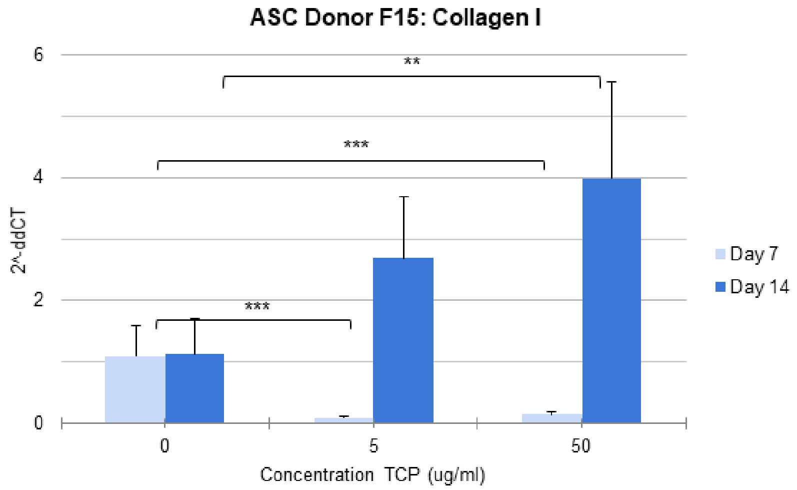
ASC Donor F15: Collagen I
2^-ddCT
6
4
2
0
**
***
***
Day 7
Day 14
0
5
50
Concentration TCP (ug/ml)

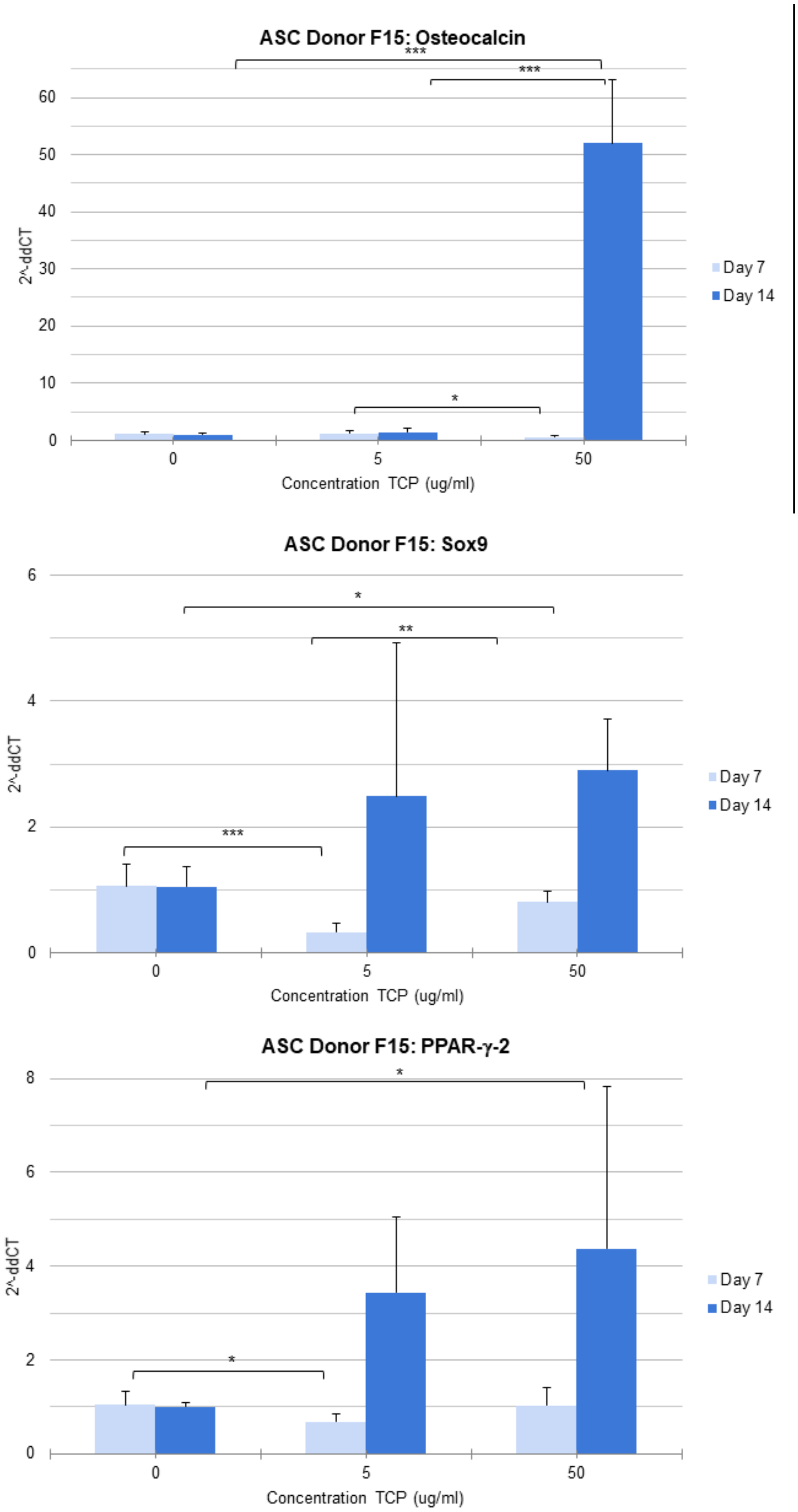

Figure S6. Average manifold induction of different genes for donor 3 (internal abbreviation F15). Experiments were carried out for three conditions and two time points, with 0, 5 or 50 ug/mL aCaP nanoparticles and at 7 or 14 days in culture, respectively. Key: TCP = amorphous calcium phosphate nanoparticles.

Table S1. The sequences of forward and reverse primers.

Gene		Sequence (5'-3')
GAPDH	forward	ACC ACA GTC CAT GCC ATC AC
	reverse	TCC ACC ACC CTG TTG CTG TA
CD31	forward	ATT GCA GTG GTT ATC ATC GGA
	reverse	CTC GTT GTT GG AGT TCA GAA GTG
CD34	forward	TGA AGC CTA GCC TGT CAC CT
	reverse	CGC ACA GCT GGA GGT CTT AT
CD73	forward	CTC CTC TCA ATC ATG CCG CT
	reverse	CCC AGG TAA TTG TGC CAT TGT
CD90	forward	TGA ATA CAG ACT GCA CCT CCC
	reverse	CTT GAC GGG TGA GGC TAG GA
CD105	forward	CAG CAG TGT CTT CCT GCA TC
	reverse	AGT TCC ACC TTC ACC GTC AC
ALP	forward	CTG GTA GTT GTT GTG AGC AT
	reverse	CCC AAA GGC TTC TTC TTG
Osteocalcin	forward	CAC TCC TCG CCC TAT TGG C
	reverse	CCC TCC TGC TTG GAC ACA AAG
Runx2	forward	GAA CCC AGA AGG CAC AGA CA
	reverse	GGC TCA GGT AGG AGG GCT
COL1	forward	TGA CGA GAC CAA GAA CTG
	reverse	CCA TCC AAA CCA CTG AAA CC
PPAR-◎-2	forward	AGG AGC AGA GCA AAG AGG
	reverse	CCT CGG ATA TGA GAA CCC
Sox 9	forward	CTC TGG AGA CTT CTG AAC GAG
	reverse	GTT CTT CAC CGA CTT CCT CCG
